# Supplementary material for: Modeling binary and graded cone cell fate patterning in the mouse retina
Source: PLoS Comput Biol. 2020 Mar 9;16(3):e1007691. doi: 10.1371/journal.pcbi.1007691 (PMC7082072; doi:10.1371/journal.pcbi.1007691)
Supplement: S1 Text — (PDF) [file pcbi.1007691.s001.pdf]

Supplementary Material for

"Modeling binary and graded cell fate patterning in the mouse retina"

Kiara C. Eldred[1], Cameron Avelis[2], Robert J. Johnston Jr.[1†], and Elijah Roberts[2†]

[1] Department of Biology, Johns Hopkins University, Baltimore, MD 21218, USA

[2] Department of Biophysics, Johns Hopkins University, Baltimore, MD 21218, USA

[†] Correspondence to:

Robert Johnston

Department of Biology, Johns Hopkins University

Mudd Hall 309, 3400 N Charles St, Baltimore, MD 21218

Ph: 410-516-2384

Email: robertjohnston@jhu.edu

or

Elijah Roberts

Department of Biophysics, Johns Hopkins University

Jenkins Hall 110, 3400 N Charles St, Baltimore, MD 21218

Ph: 410-516-2384

Email: eroberts@jhu.edu

Supplementary Text S1

1 Methods

1.1 Analysis of retina microscopy images

1.1.1 Segmentation of individual photoreceptor cells in 200X images

To identify and segment individual cone cells in the immunostained fluorescence images, we developed an image processing pipeline similar to that used previously [1]. The analysis begins finding potential cone cells by looking for connected regions in our 200X fluorescence images. Because some cells are present in only the blue (S-opsin) or green (M-opsin) channels and some cells are present in both, we combine all potential cells into a joint list for additional analysis. Algorithm 1 outlines this first step of our processing pipeline.

Algorithm 1: Extract the subimage surrounding each potential cone cell.

```
Ig,Ib = load green and blue fluorescence channels;
Ng,Nb = subtract background and normalize (Ig,Ib);
Mg,Mb = threshold images (Ng,Nb);
Og,Ob = find connected regions (Mg,Mb);
Lg,Lb = remove small regions (Og,Ob);
Fg,Fb = apply Gaussian smoothing (Lg,Lb);
Cg,Cb = find connected regions (Fg,Fb);
subimages = [];
for C in {Cg,Cb} do
    I = extract local subimage (C);
    append to subimages (I);
end
return subimages;
```

Next, we find the outline of each potential cell using an active contouring method known as

morphological snakes [2]. Because we are segmenting tens-of-thousands of individual potential cells from each image, we perform this step of the pipeline in parallel using the Biospark framework [3], which is a data intensive parallel analysis package for Python. We perform validation of the segmented boundaries before classifying the object as a cone cell.

Algorithm 2: Identify and segment any cone cells in each subimage.

```
cells = [];
parallel for I in subimages do
    P = find peak (I);
    C = active contour (I,P);
    if validate (C) then
        append to cells (C);
    end
end
```

Finally, we perform a reconciliation step in which overlapping cells are merged and/or split to obtain an estimated final segmentation for the image. The pixel indices associated with each cell are stored so that the properties of each cone cell can be later calculated. All of the scripts and Jupyter notebooks implementing our analysis pipeline are available for download from our website: `https://www.robertslabjhu.info/home/software/mouse_eye`.

1.1.2 Validation of segmentation results

We validated that our segmentation algorithm produced results similar to human annotators by comparing manually and automatically generated statistics from representative samples of our data set. We picked seven different regions and manually counted the number of S-opsin only, M-opsin only, and coexpressing cells. We then analyzed the same regions using our segmentation algorithm and obtained the automatically generated classifications.

Table S2 shows the counts of cell types from these regions from both human and computer annotations. Overall, there is excellent agreement in the relative abundance of the different cell

types. The absolute counts have some systematic difference, with the automatic segmentation typically identifying more cells than human annotators. This is mostly due to what appear to be single long cells that are split into multiple bright pieces separated by low fluorescence breaks. Human annotators tend to regard the trace as a single long cell, while the automatic segmentation tends to identify multiple smaller cells. Importantly, we do not know the true underlying cell morphology, so we cannot generally say whether the human or automatic annotators are more accurate. In any case, since the cell parts are identified correctly and the segmentation is consistent across retinas, we expect these minor difference to have no impact on our results.

1.2 Modeling of photoreceptor cells

1.2.1 Modeling cone cell fate determination and opsin expression in a retinal strip

To model the cone fate decisions in a large retinal strip we use a combination of stochastic and deterministic modeling. We start with a three-dimensional volume 5 mm long in the X dimension, 1 mm wide in the Y dimension, and 5 μm in the Z dimension representing the microenvironment of the dorsal-ventral (DV) strip. The small z dimension make this an effectively two-dimensional system and we include z below only for completeness. Within this volume we model diffusion of thyroid hormone (T3) using the deterministic diffusion equation:

$$\frac{\partial C(\boldsymbol{r},t)}{\partial t} = D\nabla^2 C(\boldsymbol{r},t), \tag{S1}$$

where $C(\boldsymbol{r},t)$ is the concentration of T3 at position $\boldsymbol{r}$ and time t, D is the diffusion coefficient used for T3, and ∇^2 is the Laplace operator ($\frac{\partial^2 C}{\partial x^2} + \frac{\partial^2 C}{\partial y^2} + \frac{\partial^2 C}{\partial z^2}$). See Table S3 for all parameter values used.

We numerically solve the diffusion partial differential equation (PDE) using a explicit finite difference method with grid spacing dx and a time step $dt = (dx^2)/(2 \cdot 6D)$, where the extra factor of 2 in the denominator ensures numerical stability. We fix the concentration at the $X = 0$ boundary to C_{hi} and at the opposite boundary to C_{lo} to establish a stationary concentration gradient in the X dimension. The Y and Z boundaries are taken to be reflective. We initialize the concentrations $C(\boldsymbol{r},0)$ according to a linear decrease in $\boldsymbol{r}$ to follow to the boundary conditions.

Within the microenvironment, we place 23,760 individual photoreceptor cells spaced on a hexagonal grid spanning the X-Y plane, with center-to-center distance $d_{cell-cell}$ and with a radius r_{cell}. Each cell is modeled independently using the chemical master equation (CME) describing the probability to have a particular count of each species:

$$\frac{dP_t(\boldsymbol{x})}{dt} = \boldsymbol{A} P_t(\boldsymbol{x}), \tag{S2}$$

where $P_t(\boldsymbol{x})$ is the probability for a cell to have a particular state vector $\boldsymbol{x}$ giving the count for each chemical species and $\boldsymbol{A}$ is a transition matrix describing all of the reactions between the chemical species.

Within each photoreceptor cell a series of reactions describing the fate of the cell and also opsin expression take place. First, cells contain thyroid hormone receptors THRβ2. T3 can bind reversibly to THRβ2 to switch it to an activated state THRβ2*:

$$\mathrm{T3} + \mathrm{THR}\beta 2 \underset{k_r^{thrb}}{\overset{k_f^{thrb}}{\rightleftharpoons}} \mathrm{THR}\beta 2^*. \tag{S3}$$

Note that T3 also diffusing across the PDE microenvironment and the value of T3 is synchronized between the PDE and CME models. The synchronization procedure is discussed below.

Next, the cells switch between three cell fates. FD(U) cells are undifferentiated and do not express opsins, FD(S) cells occupy an S-only cone cell fate with high expression of only S-opsin, and FD(C) cells are typical cone cells that express some combination of S- and M-opsin depending on various factors. The transition between cone cell fates in real cells depends on a number of unknown fate determining steps. Little is known about these steps, but the fate decisions appear to be stable. Therefore, we model photoreceptor fate decision-making as barrier crossing process with a variable number of cooperative steps n.

The FD(U) $\leftrightarrow$ FD(S) transition is described by

$$\mathrm{FD(U)} \xrightleftharpoons[0.5^i \cdot k_r^{fds}]{H'(\mathrm{THR}\beta 2^*)\cdot k_f^{fds}} \mathrm{FD(U{\rightarrow}^{i+1}S)} \qquad \text{for } i = 0, \tag{S4}$$

$$\mathrm{FD(U{\rightarrow}^{i}S)} \xrightleftharpoons[0.5^i \cdot k_r^{fds}]{2^i \cdot k_f^{fds}} \mathrm{FD(U{\rightarrow}^{i+1}S)} \qquad \text{for } i > 0 \text{ and } i < n-1, \tag{S5}$$

$$\mathrm{FD(U{\rightarrow}^{i}S)} \xrightleftharpoons[0.5^i \cdot k_r^{fds}]{2^i \cdot k_f^{fds}} \mathrm{FD(S)} \qquad \text{for } i = n-1. \tag{S6}$$

Here, the syntax FD(U$\rightarrow^i$S) denotes a cell that has progressed i steps along the path from the FD(U) to FD(S) fate. Also, H' is an inhibiting Hill-like kinetic function defined by

$$H'(X) = k_{lo} + \frac{(k_{hi} - k_{lo})k_m^h}{k_m^h + x^h}, \tag{S7}$$

with k_{lo} and k_{hi} the lower and upper limits of the kinetic process, respectively, k_m the midpoint of the transition, and h the Hill exponent giving the cooperativity of the transition. Likewise, the FD(U) $\leftrightarrow$ FD(C) transition is described by

$$\mathrm{FD(U)} \xrightleftharpoons[0.5^i \cdot k_r^{fdc}]{2^i \cdot k_f^{fdc}} \mathrm{FD(U{\rightarrow}^{i+1}C)} \qquad \text{for } i = 0, \tag{S8}$$

$$\mathrm{FD(U{\rightarrow}^{i}C)} \xrightleftharpoons[0.5^i \cdot k_r^{fdc}]{2^i \cdot k_f^{fdc}} \mathrm{FD(U{\rightarrow}^{i+1}C)} \qquad \text{for } i > 0 \text{ and } i < n-1, \tag{S9}$$

$$\mathrm{FD(U{\rightarrow}^{i}C)} \xrightleftharpoons[0.5^i \cdot k_r^{fdc}]{2^i \cdot k_f^{fdc}} \mathrm{FD(C)} \qquad \text{for } i = n-1. \tag{S10}$$

Then, both S-opsin and M-opsin proteins can be expressed by photoreceptor cells, depending on the cell type and the local concentration of T3. We model opsin expression using the following kinetic equations

$$\varnothing \xrightarrow{k_f^{ss}} \mathrm{S} \qquad \text{if FD(P)}, \tag{S11}$$

$$\varnothing \xrightarrow{[H'(\mathrm{THR}\beta 2^*) + H(\mathrm{THR}\beta 2)]\cdot k_f^{cs}} \mathrm{S} \qquad \text{if FD(C)}, \tag{S12}$$

$$\varnothing \xrightarrow{H(\mathrm{THR}\beta 2^*)\cdot k_f^{cm}} \mathrm{M} \qquad \text{if FD(C)}, \tag{S13}$$

H is an activating Hill-like kinetic function

$$H(X) = k_{lo} + \frac{(k_{hi} - k_{lo})x^h}{x_m^h + x^h}. \tag{S14}$$

Finally, both opsins can be degraded according to

$$\mathrm{S} \xrightarrow{k_{deg}^s} \varnothing, \tag{S15}$$

$$\mathrm{M} \xrightarrow{k_{deg}^m} \varnothing. \tag{S16}$$

We initialize each cell at $t = 0$ to the FD(U) state with a copy number of THRβ2 proteins independently sampled from a Gaussian distribution with a mean concentration of μ_{thrb} and a variance of σ_{thrb}^2. The number of T3 molecules available to the cell is initialized according to the T3 concentration at the cell's D-V position. Likewise, the fraction of activated THRβ2 is initialized to its equilibrium value according to the cell's D-V position. All opsin counts are initialized to zero. We then model the stochastic time evolution of each cell using the standard Gillespie stochastic simulation algorithm (SSA) [4, 5].

1.2.2 Microenvironment modeling of combined PDE and CME dynamics

Since we are performing two parallel simulations, PDE and CME, we need to partition the molecules between them. Each cell has a volume smaller than a PDE subvolume and each cell is assumed to be completely contained within a single subvolume. We initialize the T3 molecule count in the extracellular space of each cell to be the rounded number of molecules corresponding to the cell's extracellular volume multiplied by the PDE subvolume's concentration. In this way T3 molecules are represented in each simulation.

To integrate the PDE and CME dynamics, we implemented a parallel time-stepping approach. We divide time into discrete synchronization intervals Δt and evolve overall time according to

$$t_{i+1} = t_i + \Delta t. \tag{S17}$$

During each Δt we update the state of each cell using the SSA and the concentration of each

PDE subvolume using a finite difference algorithm. The loss or gain in the number of extracellular T3 molecules in the stochastic simulation during the time step is tracked. This gain or loss is converted into a concentration flux and applied to the PDE subvolume during the next time step. Simultaneously, the number of extracellular T3 molecules in the stochastic model is reset for the next time step according to the new subvolume concentration. Our method conserves mass and has good error characteristics as long as the T3 flux at each time step is of the order of a few molecules. The method is implemented as the "microenvironment" solver in our LMES software and is freely available on our website: `https://www.robertslabjhu.info/home/software/lmes`.

1.2.3 Parameterization of retinal strip microenvironment model

We parameterized our model by globally fitting the model parameters to five different dorsal-ventral (D-V) data sets: (1) the fraction of all cells expressing M-opsin, (2) the fraction of all cells expressing S-opsin, (3) the fraction of FD(S) cells expressing only S-opsin, (4) the per cell M-opsin expression level, and (5) the per cell S-opsin expression level. Because our data were collected from multiple retinas, we first fit the raw data to functions that we could use to describe a hypothetical mean retina.

We describe the fraction of cells in the various subfates as (modified) Hill-like functions. The fraction of cells expressing S-opsin as a function of D-V position x is described by:

$$F_S(x) = m \cdot x + b + [1 - (m \cdot x + b)] \cdot \frac{x^h}{x_{mid}^h + x^h}, \tag{S18}$$

where m and b are the slope and x-intercept of a baseline fraction, respectively, x_{mid} is the midpoint of the transition, and h is the Hill coefficient. Likewise, the fraction of M-opsin expressing cells is given by:

$$F_M(x) = \frac{x_{mid}^h}{x_{mid}^h + x^h}, \tag{S19}$$

and the fraction of FD(S) cells is given by:

$$F_{FDS}(x) = F_{min} + (F_{max} - F_{min}) \cdot \frac{x^h}{x_{mid}^h + x^h}. \tag{S20}$$

Figures S2+S4 show the fits of these functions to the raw data for the various retinas. We then took the mean of the various parameters to construct a hypothetical mean retina. Figure S14 shows the fraction of cells in these states as a function of D-V position in our mean retina.

We describe the mean per cell expression level of M- and S-opsin as piecewise linear functions with a low and a high limit separated by a biphasic region with two different slopes:

$$I(x) = y_0 \quad \text{if } x < x_0, \tag{S21}$$

$$= y_0 + m_1 \cdot (x - x_0) \quad \text{if } x_0 < x < x_1, \tag{S22}$$

$$= y_0 + m_1 \cdot (x_1 - x_0) + m_2 \cdot (x - x_1) \quad \text{if } x_1 < x < x_2, \tag{S23}$$

$$= y_0 + m_1 \cdot (x_1 - x_0) + m_2 \cdot (x_2 - x_1) + y_1 \quad \text{if } x > x_2. \tag{S24}$$

Here, y_0 and y_1 are the left and right baselines, x_0, x_1, and x_2 are the D-V points where the slope changes, and m_1 and m_2 are the two slopes. Figure S8 shows the fit to the M- and S-opsin expression for the various experimental retinas. Figure S14 shows the values of these functions for our hypothetical mean retina.

Once we had a hypothetical mean retina, we used it to parameterize our model. We derived expressions for the mean value of the various observables as a function of D-V position by solving the deterministic system of ordinary differential equations described by Equations S3-S16. We then used non-linear least squares with Nelder-Mead minimization to globally optimize the parameters using all five data sets. Table S3 gives the best fits values for the free parameters and Figure S15 shows a comparison of the deterministic model calculated using the best-fit parameters against our hypothetical mean retina.

References

[1] Anderson, C, Reiss, I, Zhou, C, Cho, A, Siddiqi, H, Mormann, B, Avelis, CM, Deford, P, Bergland, A, Roberts, E, Taylor, J, Vasiliauskas, D, Johnston, RJ (2017) Natural variation in stochastic photoreceptor specification and color preference in Drosophila. *eLife* 6:e29593.

[2] Mrquez-Neila, P, Baumela, L, Alvarez, L (2014) A morphological approach to curvature-based evolution of curves and surfaces. *IEEE Transactions on Pattern Analysis and Machine Intelligence* 36:2–17.

[3] Klein, M, Sharma, R, Bohrer, CH, Avelis, CM, Roberts, E (2017) Biospark: scalable analysis of large numerical datasets from biological simulations and experiments using Hadoop and Spark. *Bioinformatics* 33:303–5.

[4] Gillespie, DT (1977) Exact stochastic simulation of coupled chemical reactions. *J. Phys. Chem.* 81:2340–61.

[5] Roberts, E, Stone, JE, Luthey-Schulten, Z (2013) Lattice Microbes: High-performance stochastic simulation method for the reaction-diffusion master equation. *J. Comput. Chem.* 34:245–55.
