# Supplementary material for: Modeling binary and graded cone cell fate patterning in the mouse retina
Source: PLoS Comput Biol. 2020 Mar 9;16(3):e1007691. doi: 10.1371/journal.pcbi.1007691 (PMC7082072; doi:10.1371/journal.pcbi.1007691)
Supplement: S1 Table — (PDF) [file pcbi.1007691.s002.pdf]

Table S1: Retina image names and genotypes.

Retina	Filename	Genotype	Num. Cells
R07	170505_2M_WT_F1_Left_20x_Stitch_MIP	WT	65467
R08	170505_2M_WT_F1_Right_20x-Stitch-MIP	WT	50437
R09	170505_2M_WT_F2_Left_20x-Stitch-MIP	WT	34381
R22	170817_pregnantMALE_CONTROL_BL6_1_20x-Stitch-MIP_c1+2	WT	40660
R25	171026_WT_F1_Left_20x-Stitch-MIP	WT	29831
R26	171026_WT_F1_Right_20x-Stitch-MIP	WT	29962
R31	180123_ThrB2_KO_4M_DV_Right_slide1_20x_Stitch-MIP	ΔTHRβ2	10781
R32	180123_ThrB2_KO_4M_DV_Right_slide2_20x-Stitch-MIP	ΔTHRβ2	10508
R33	180123_ThrB2_KO_4M_whole_Right_slide1_20X-Stitch-MIP	ΔTHRβ2	15570
