# Supplementary material for: Modeling binary and graded cone cell fate patterning in the mouse retina
Source: PLoS Comput Biol. 2020 Mar 9;16(3):e1007691. doi: 10.1371/journal.pcbi.1007691 (PMC7082072; doi:10.1371/journal.pcbi.1007691)
Supplement: S2 Table — (PDF) [file pcbi.1007691.s003.pdf]

Table S2: The number of cells identified per subtype by hand (H) and computer (C) annotation.

Retina Section Filename	S- & M-opsin (H/C)	S-only (H/C)	M-only (H/C)
171026_WT_F1_Left_20x-Stitch-MIP-DORSAL	13/23	4/6	584/753
171026_WT_F2p_Left_780_20x-Stitch-MIP-VENTRAL	267/313	82/70	2/1
171026_WT_F2p_Left_780_20x-Stitch-MIP-CENTER	186/277	20/26	0/1
171026_WT_F2p_Left_780_20x-Stitch-MIP-DORSAL	4/5	10/13	427/377
171026_WT_F2p_Right_800_20x-Stitch-MIP-VENTRAL	380/783	198/303	0/0
171026_WT_F2p_Right_800_20x-Stitch-MIP-CENTER	515/1060	64/81	8/1
171026_WT_F2p_Right_800_20x-Stitch-MIP-DORSAL	17/29	3/4	259/264
