# Supplementary material for: Modeling binary and graded cone cell fate patterning in the mouse retina
Source: PLoS Comput Biol. 2020 Mar 9;16(3):e1007691. doi: 10.1371/journal.pcbi.1007691 (PMC7082072; doi:10.1371/journal.pcbi.1007691)
Supplement: S3 Table — (PDF) [file pcbi.1007691.s004.pdf]

Table S3: Parameters used in the microenvironment model.

Name	Value	Fit	Description
Simulation Setup			
$strip\_width$	1000×10^{-6} m		Retinal strip width
$strip\_height$	5000×10^{-6} m		Retinal strip height
dx	5×10^{-6} m		PDE lattice spacing
dt	8.33×10^{-2} s		PDE time step
$d_{cell-cell}$	15×10^{-6} m		Center-to-center spacing between cells
r_{cell}	5×10^{-6} m		Cell radius
t_{sync}	1.0×10^{0} s		Synchronization time between PDE and CME simulations
PDE Model			
D	1×10^{-10} $m^2\,s^{-1}$		T3 diffusion coefficient
C_{hi}	1×10^{-7} M		T3 dorsal concentration
C_{lo}	1×10^{-9} M		T3 ventral concentration
CME Model - THRβ2 activity			
μ_{thrb}	1×10^{-8} M		Mean of the cellular THRβ2 concentration
σ^2_{thrb}	$1.0 \cdot \mu_{thrb}$ M^2		Variance of the cellular THRβ2 concentration
k_D^{thrb}	1.51×10^{-7} M	Y	THRβ2-T3 equilibrium dissociation constant
k_f^{thrb}	1×10^{6} $M^{-1}s^{-1}$		THRβ2-T3 kinetic on rate
k_r^{thrb}	$k_D^{thrb} \cdot k_f^{thrb}$ s^{-1}		THRβ2-T3 kinetic off rate
CME Model - fate determination			
k_f^{fds}	$10\,s^{-1}$		Forward rate for FD(S) regulation
k_{lo}^{fds}	9.72×10^{-4}	Y	Lower limit for FD(S) regulation
k_{hi}^{fds}	2.41×10^{-1}	Y	Upper limit for FD(S) regulation
k_m^{fds}	4.37×10^{2} molecules	Y	Midpoint of THRβ2* for FD(S) regulation
h^{fds}	3.61×10^{0}	Y	Hill exponent for FD(S) regulation
k_r^{fds}	$100\,s^{-1}$		Reverse rate for FD(S) regulation
k_f^{fdc}	$10\,s^{-1}$		Forward rate for FD(C) regulation
k_r^{fdc}	$100\,s^{-1}$		Reverse rate for FD(C) regulation
CME Model - opsin expression			
k_{deg}^{s}	$0.01\,s^{-1}$		S-opsin degradation rate
k_{deg}^{m}	$0.01\,s^{-1}$		M-opsin degradation rate
k_{eq}^{ss}	2×10^{-10} M		FD(S) S-opsin equilibrium concentration
k_f^{ss}	$k_{eq}^{ss} \cdot k_{deg}^{s}$ $M\,s^{-1}$		FD(S) S-opsin expression rate
k_{eq}^{cs}	2×10^{-10} M		FD(C) S-opsin equilibrium concentration
k_f^{cs}	$k_{eq}^{cs} \cdot k_{deg}^{s}$ $M\,s^{-1}$		Forward rate for FD(C) S-opsin expression
k_{lo1}^{cs}	3.05×10^{-4}	Y	Lower limit for FD(C) S-opsin expression
k_{hi1}^{cs}	6.44×10^{-1}	Y	Upper limit for FD(C) S-opsin expression
k_{m1}^{cs}	6.87×10^{2} molecules	Y	Midpoint of THRβ2* for S-opsin expression
h_1^{cs}	1.40×10^{1}	Y	Hill exponent for FD(C) S-opsin expression
k_{lo2}^{cs}	1.09×10^{-3}	Y	Lower limit for FD(C) S-opsin expression
k_{hi2}^{cs}	7.45×10^{-1}	Y	Upper limit for FD(C) S-opsin expression
k_{m2}^{cs}	2.66×10^{3} molecules	Y	Midpoint of THRβ2 for FD(C) S-opsin expression
h_2^{cs}	1.39×10^{1}	Y	Hill exponent for FD(C) S-opsin expression
k_{eq}^{cm}	2×10^{-10} M		FD(C) M-opsin equilibrium concentration
k_f^{cm}	$k_{eq}^{cm} \cdot k_{deg}^{m}$ $M\,s^{-1}$		Forward rate for FD(C) M-opsin expression
k_{lo}^{cm}	2.34×10^{-14}	Y	Lower limit for FD(C) M-opsin expression
k_{hi}^{cm}	1.26×10^{0}	Y	Upper limit for FD(C) M-opsin expression
k_m^{cm}	6.17×10^{2} molecules	Y	Midpoint of THRβ2* for FD(C) M-opsin expression
h^{cm}	6.60×10^{-1}	Y	Hill exponent for FD(C) M-opsin expression
