# Supplementary figures and images for: Modeling binary and graded cone cell fate patterning in the mouse retina
Source: PLoS Comput Biol. 2020 Mar 9;16(3):e1007691. doi: 10.1371/journal.pcbi.1007691 (PMC7082072; doi:10.1371/journal.pcbi.1007691)

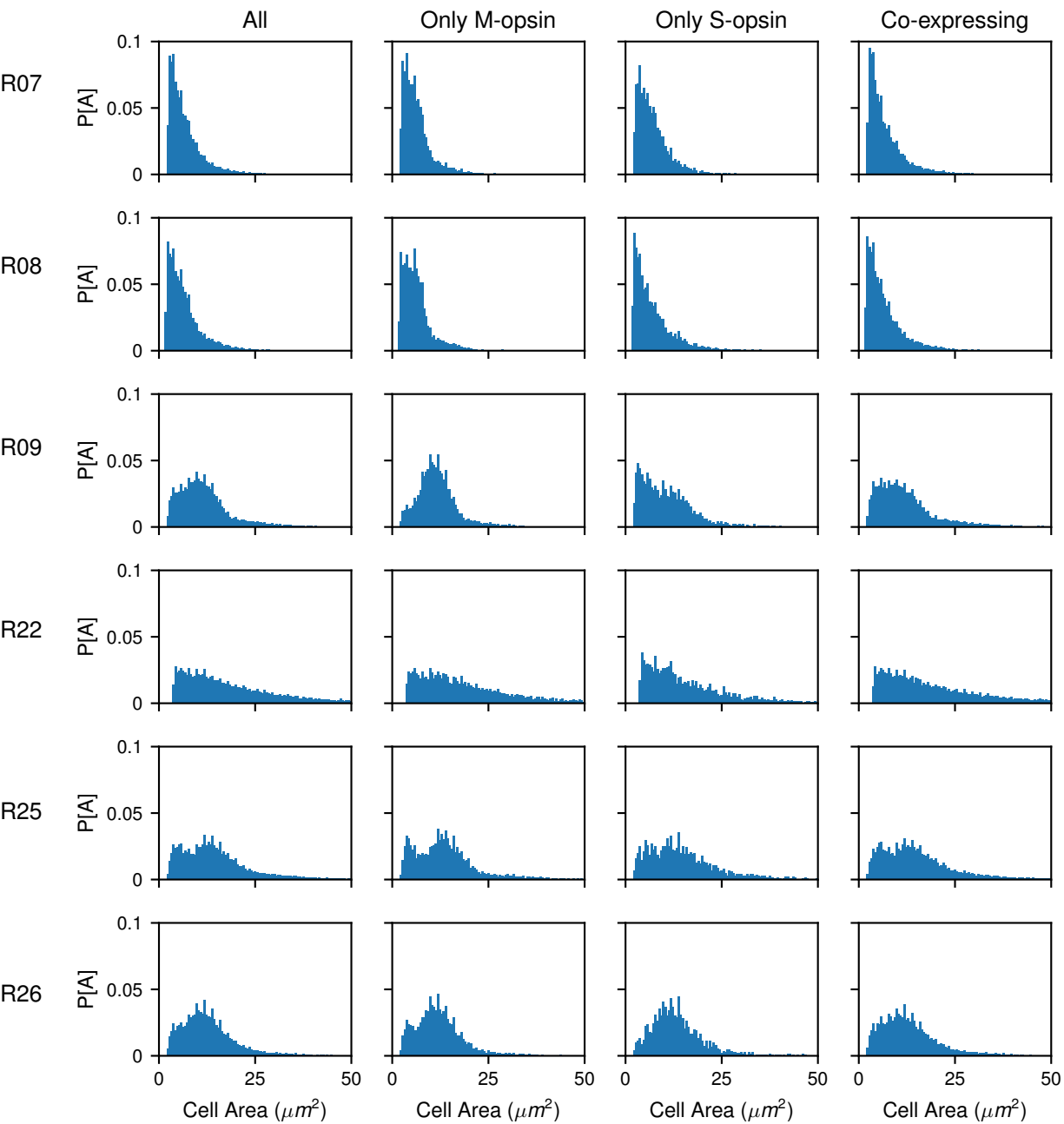

Supplement: S1 Fig — The probability distribution for the outer segment area is shown for each expression class. Columns shows different expression classes, as labeled. Rows show different retinas (RXX). No systematic differences were observed between the classes, but note that retina-to-retina variability in outer segment area is present due to variations in mounting and subsequently the angle of image capture for cone cell outer segments. (PDF) [file pcbi.1007691.s005.pdf]

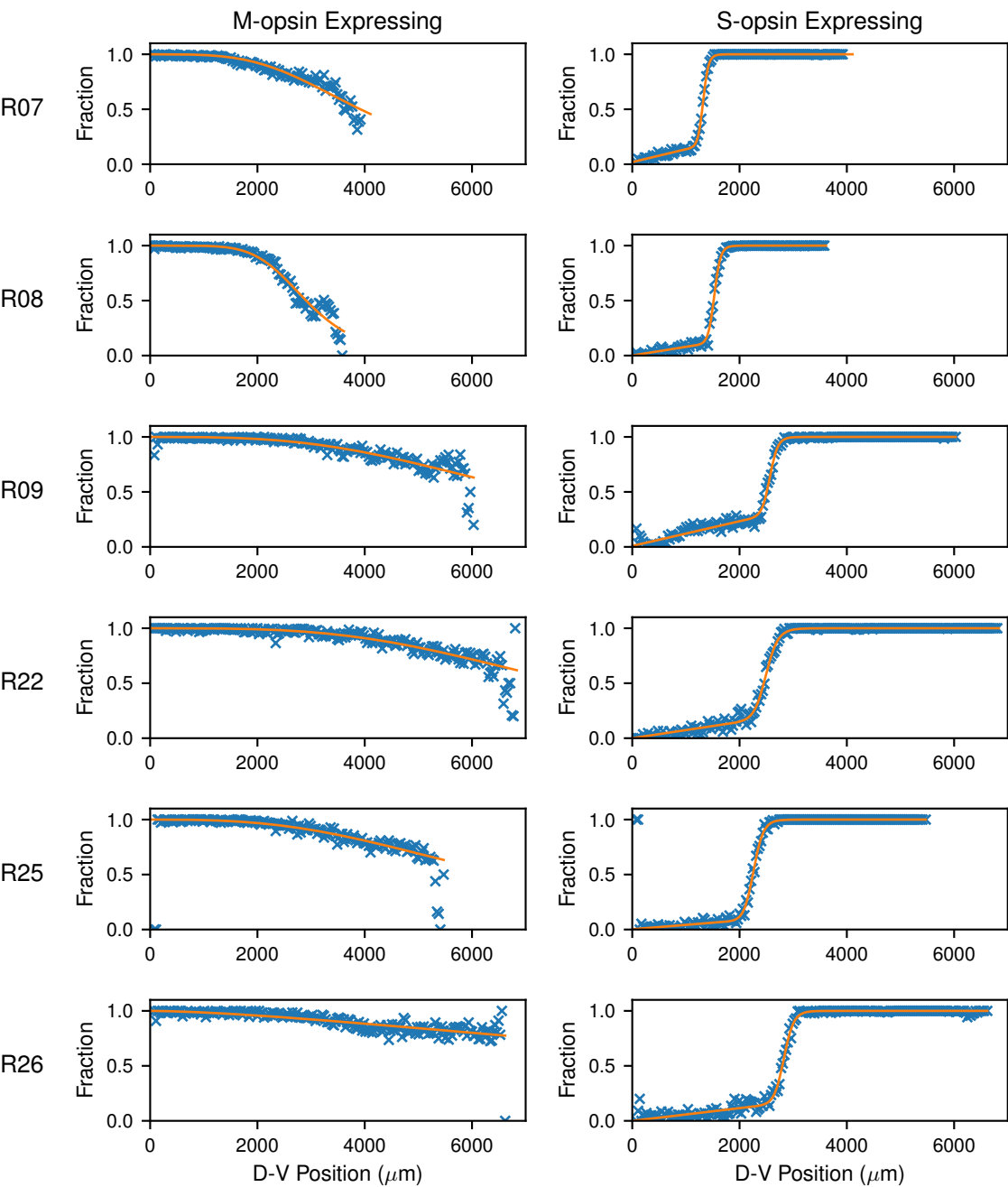

Supplement: S2 Fig — Fraction of cells expressing (left) M-opsin and (right) S-opsin by position along the D-V axis. The data from the microscopy analysis (x) are overlaid with the best fit (line) to a fitting function (see text). Rows show different retinas (RXX). (PDF) [file pcbi.1007691.s006.pdf]

# S-opsin-only Expressing

R07

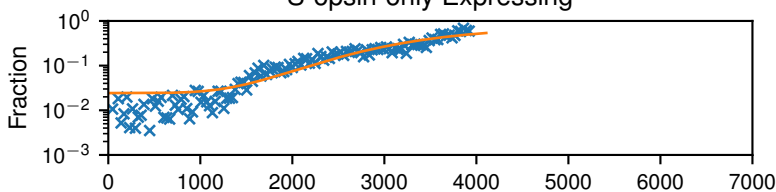

R08

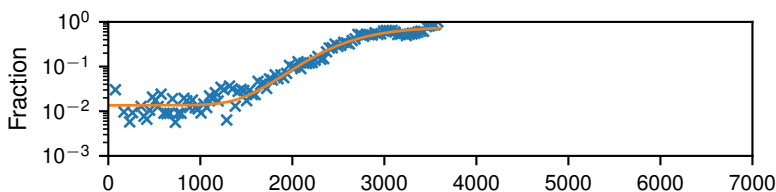

R09

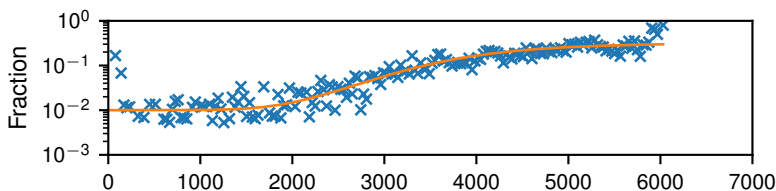

R22

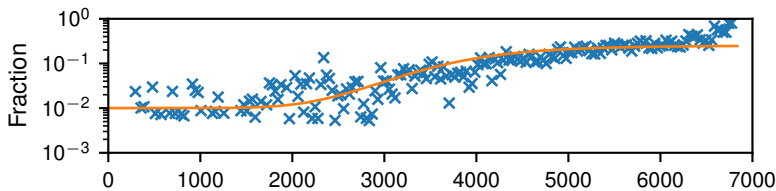

R25

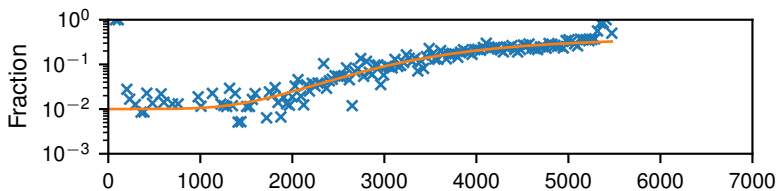

R26

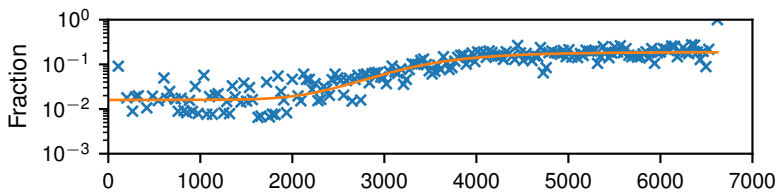

D-V Position ( $\mu\text{m}$ )

Supplement: S4 Fig — Fraction of cells expressing only S-opsin by position along the D-V axis. The data from the microscopy analysis (x) are overlaid with the best fit (line) to a fitting function (see text). Rows show different retinas (RXX). (PDF) [file pcbi.1007691.s008.pdf]

R07

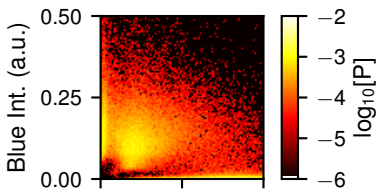

R08

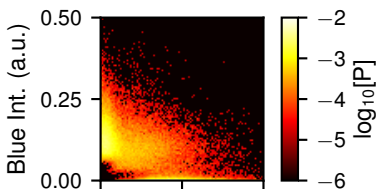

R09

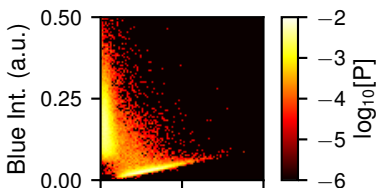

R22

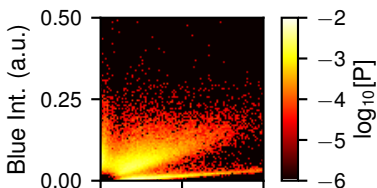

R25

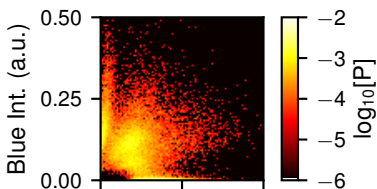

R26

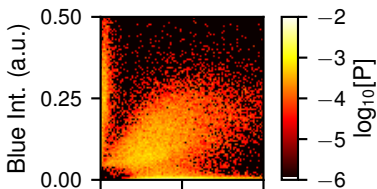

0 0.25 0.5  
Green Int. (a.u.)

Supplement: S5 Fig — Joint probability distributions for the abundance of S-opsin (blue intensity) and M-opsin (green intensity) in cells. Rows show different retinas (RXX). Colors range from log_10[P] = −2 (white/yellow) to log_10[P] = −6 (red/black). (PDF) [file pcbi.1007691.s009.pdf]

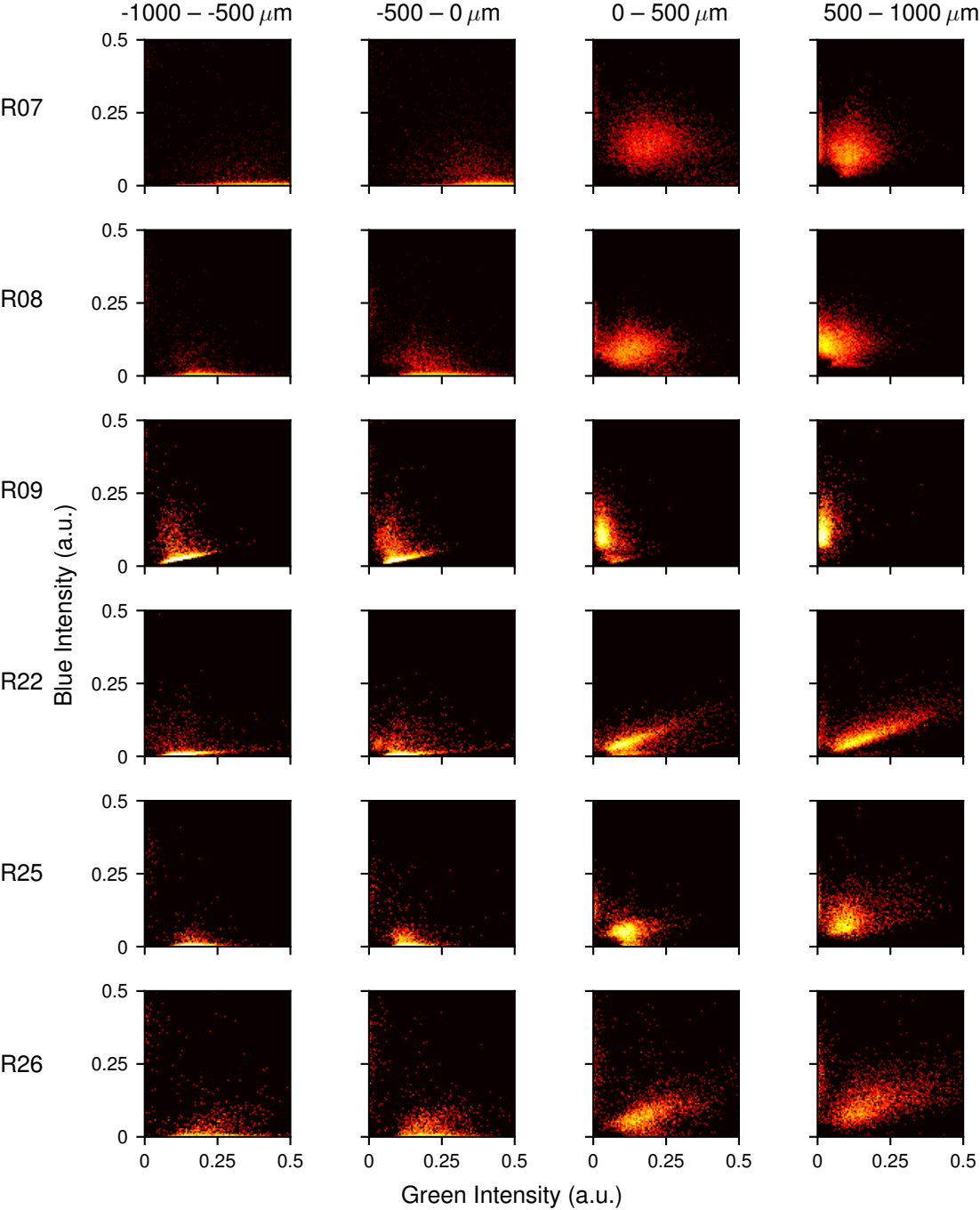

Supplement: S6 Fig — Joint probability distributions for the abundance of S-opsin (blue intensity) and M-opsin (green intensity) in cells. Columns show cells binned from four different regions according to distance from the transition midpoint. Rows show different retinas (RXX). Colors range from log_10[P] = −2 (white/yellow) to log_10[P] = −4 (red/black). (PDF) [file pcbi.1007691.s010.pdf]

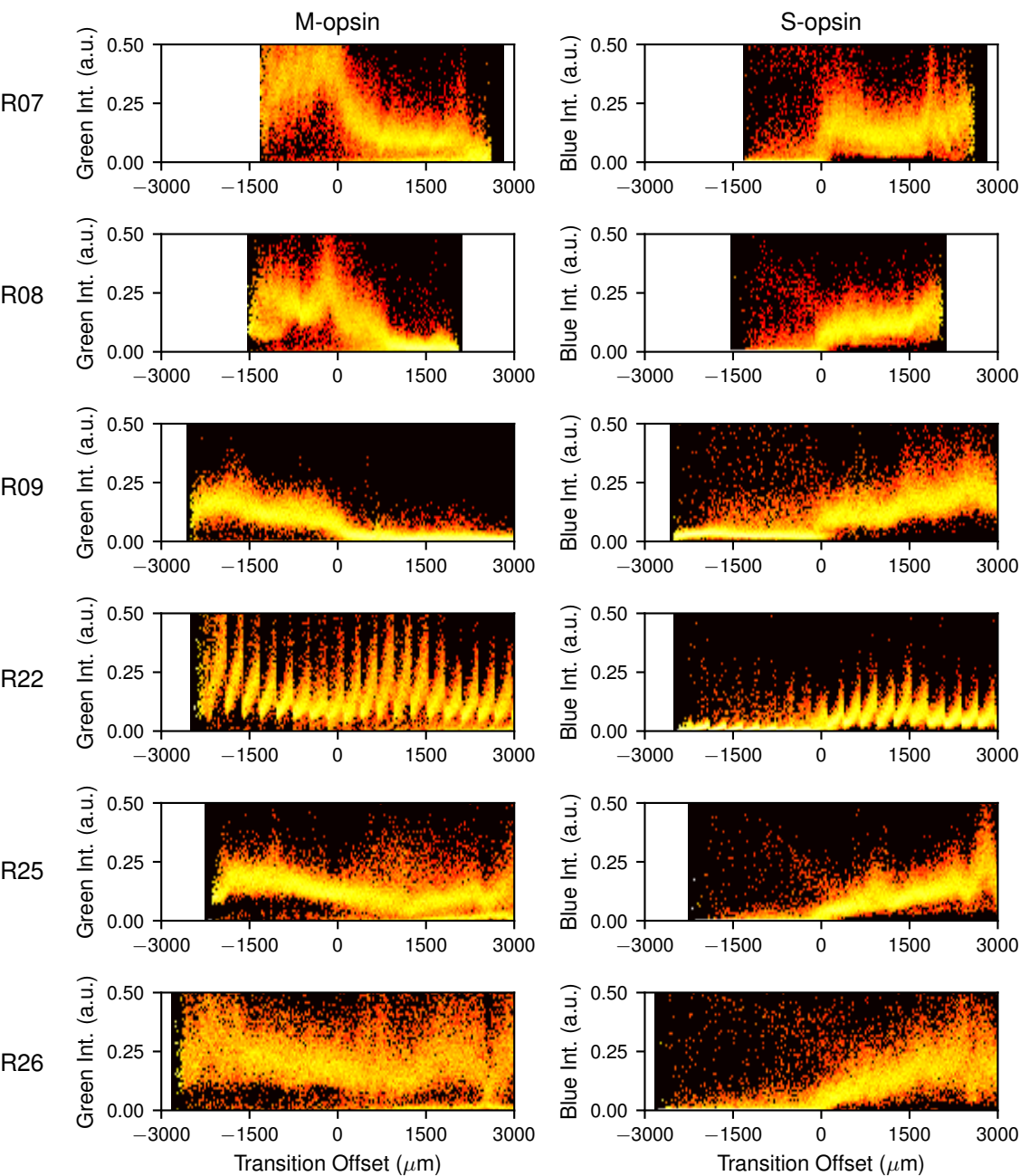

Supplement: S7 Fig — Probability distribution for the abundance of (left) M-opsin and (right) S-opsin in cells by distance from the transition midpoint. Rows show different retinas (RXX). Colors range from log_10[P] = 0 (white/yellow) to log_10[P] = −4 (red/black). (PDF) [file pcbi.1007691.s011.pdf]

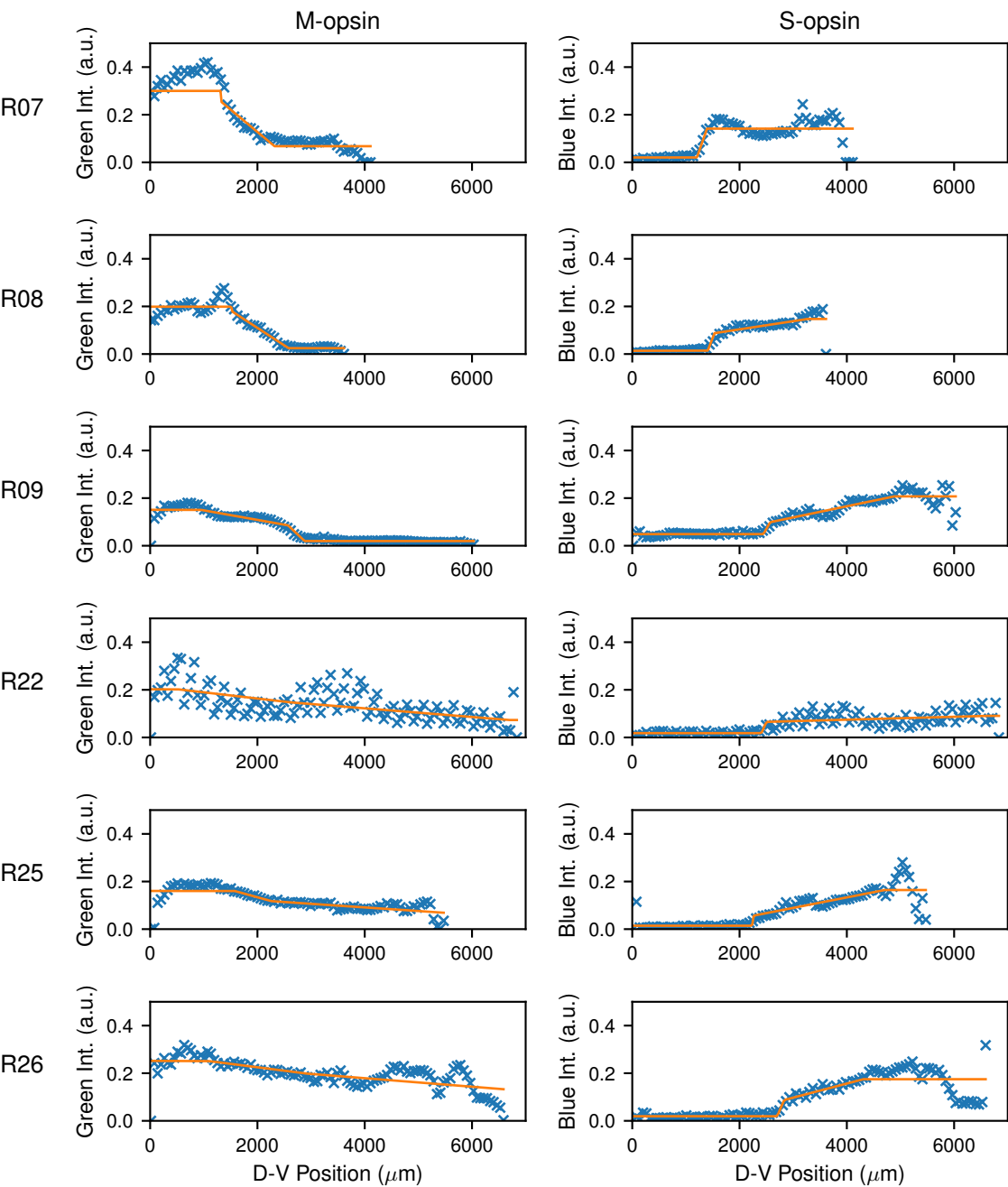

Supplement: S8 Fig — Mean intensity in all cells of (left) M-opsin and (right) S-opsin by position along the D-V axis. The data from the microscopy analysis (x) are overlaid with the best fit (line) to a fitting function (see text). Rows show different retinas (RXX). (PDF) [file pcbi.1007691.s012.pdf]

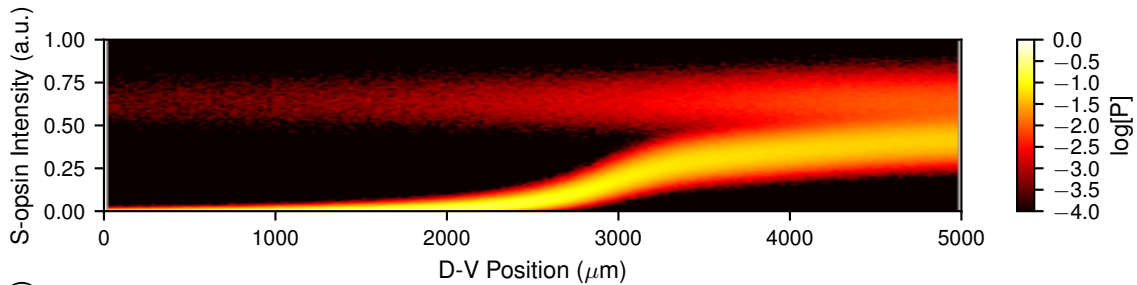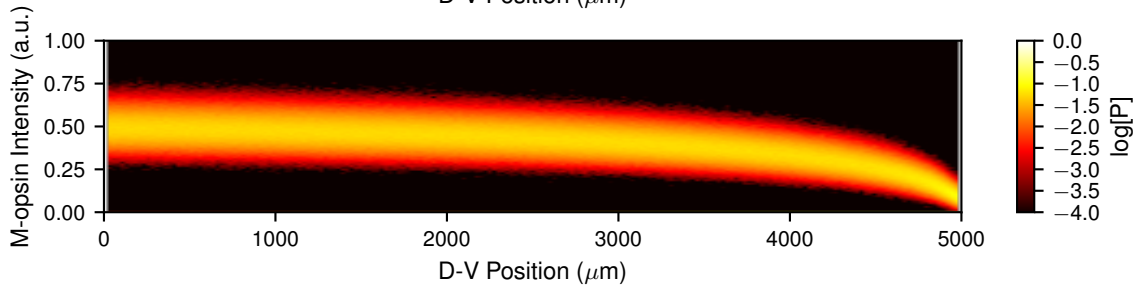

Supplement: S10 Fig — Probability distribution of the abundance of S-opsin (blue intensity) and M-opsin (green intensity) in cells along the D-V axis from numerical simulations of the model. Distributions were computed from 100 independent simulations. (PDF) [file pcbi.1007691.s014.pdf]

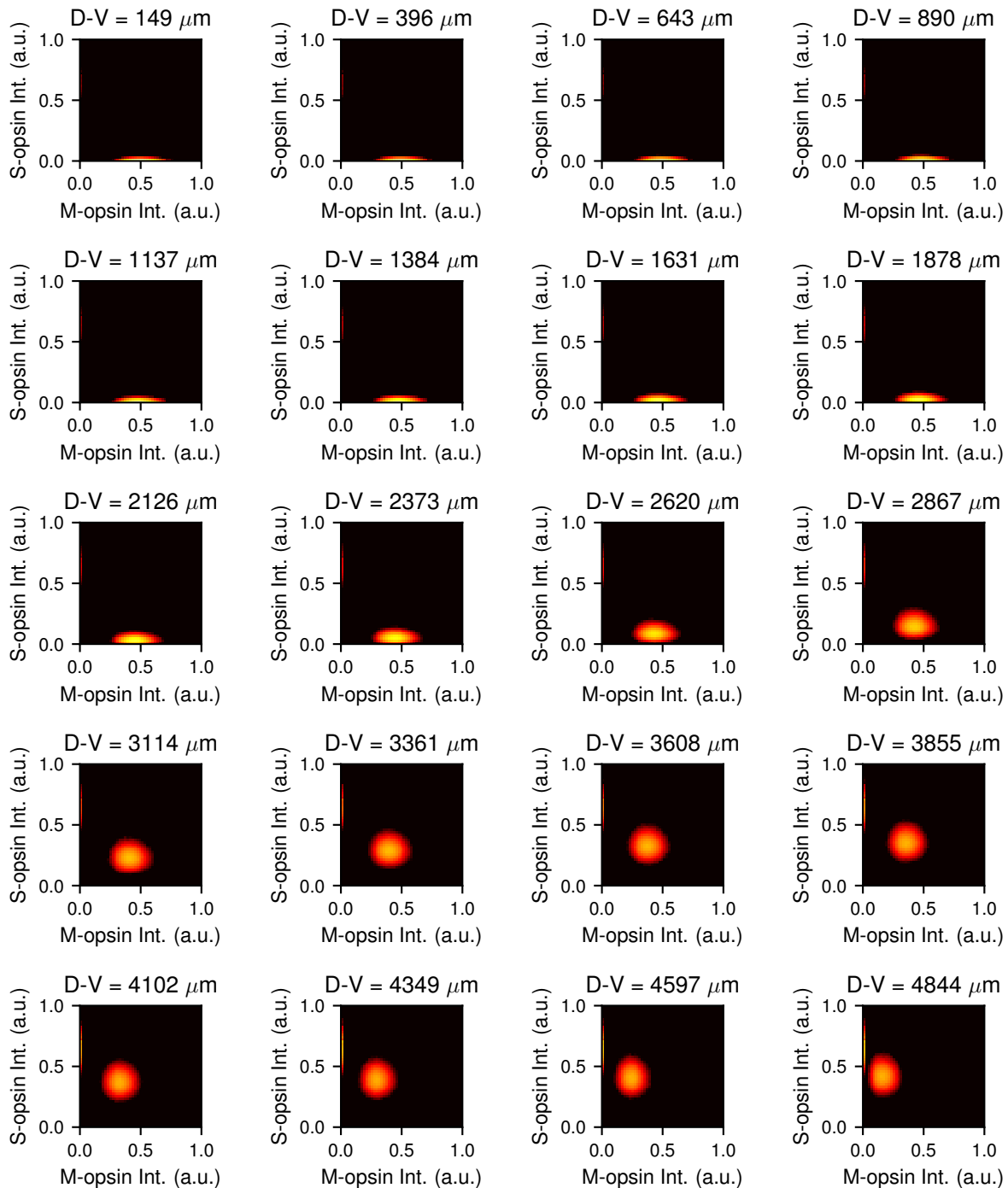

Supplement: S11 Fig — Joint probability distributions for the abundance of S-opsin (blue intensity) and M-opsin (green intensity) in cells located in ∼250μm wide bins along the D-V axis. Colors range from log_10[P] = −2 (white/yellow) to log_10[P] = −5 (red/black). Distributions were computed from 100 independent simulations. The low density tails leading to 0,0 are from cells that were sampled during the process of switching phenotypes. (PDF) [file pcbi.1007691.s015.pdf]

R31  
CELL

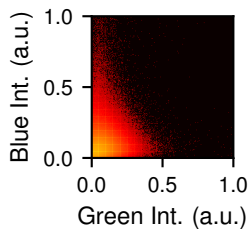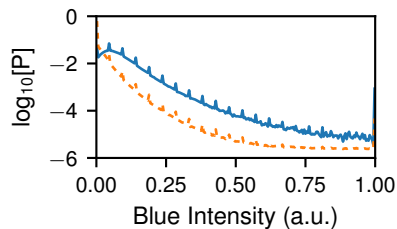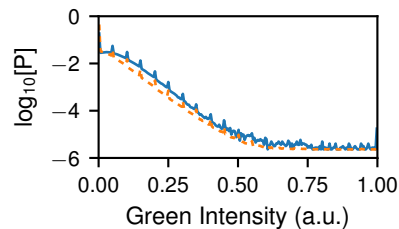

R31  
BG

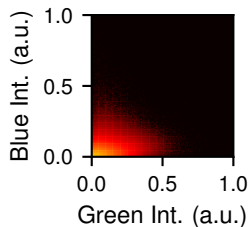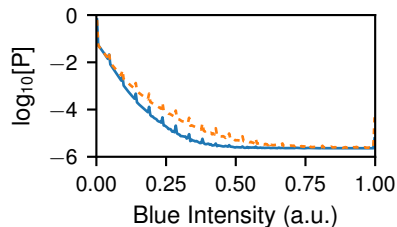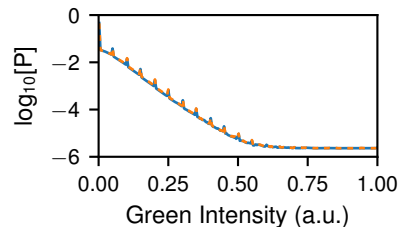

R32  
CELL

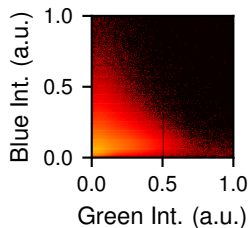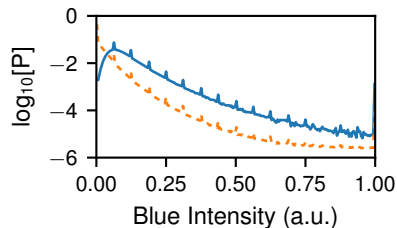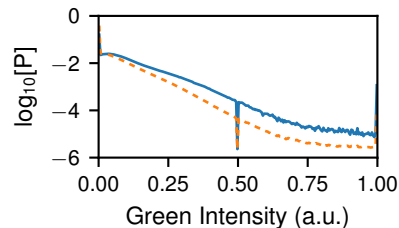

R32  
BG

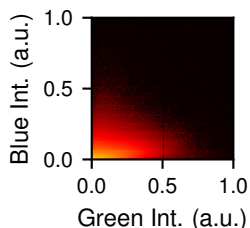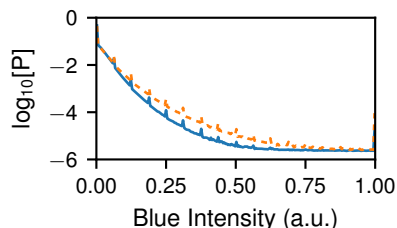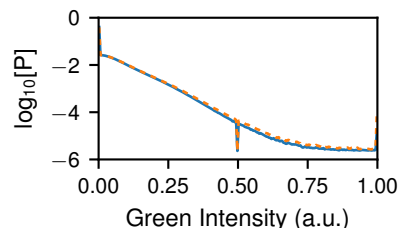

R33  
CELL

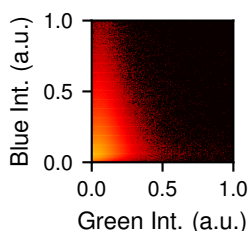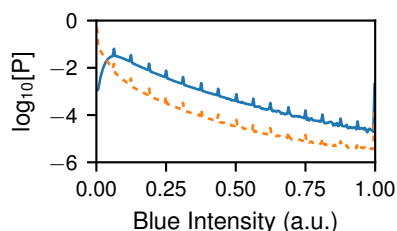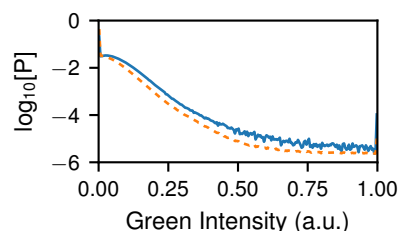

R33  
BG

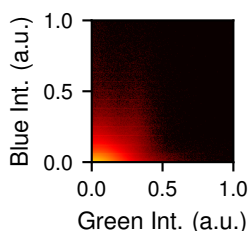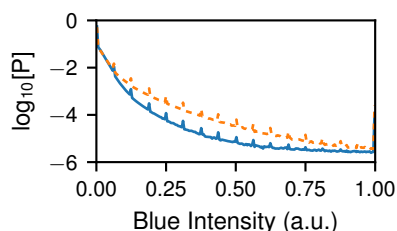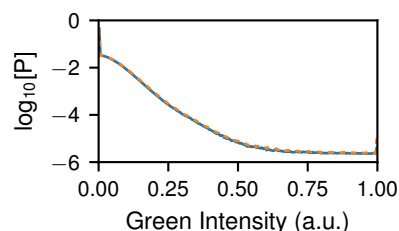

Supplement: S12 Fig — (left) Joint probability distribution of the blue and green intensity of pixels located either inside of cell boundaries (RXX CELL) or the background outside of cells (RXX BG) as indicated. Colors range from log_10[P] = 0 (white/yellow) to log_10[P] = −8 (red/black). (center) Probability for a pixel of the indicated type to have a particular blue intensity (solid line) compared with the distribution for all pixels (dashed line). (right) The same for green intensity. ΔTHRβ2 cells do not exhibit green expression above background. (PDF) [file pcbi.1007691.s016.pdf]

R31

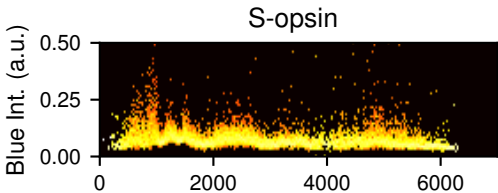

R32

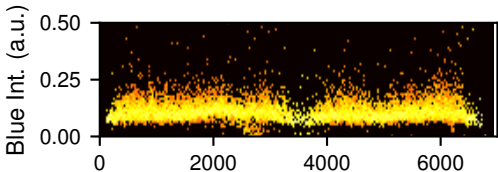

R33

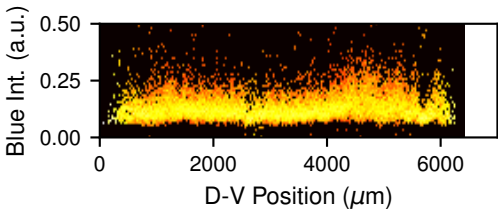

Supplement: S13 Fig — Probability distribution for the abundance of S-opsin in cells by distance along the D-V axis. Rows show different ΔTHRβ2 retinas (RXX). Colors range from log_10[P] = 0 (white/yellow) to log_10[P] = −4 (red/black). (PDF) [file pcbi.1007691.s017.pdf]

M-opsin Expressing

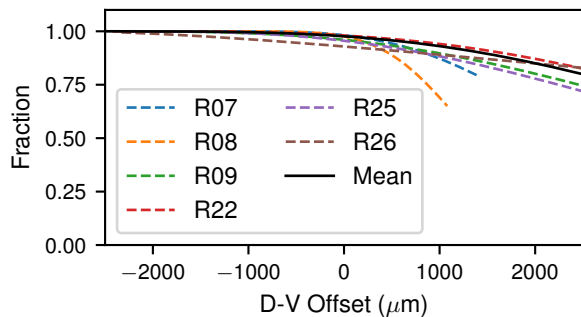

S-opsin Expressing

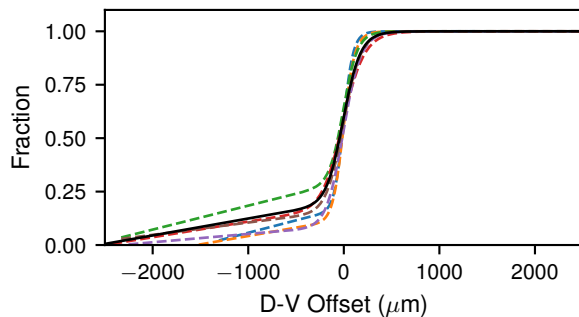

Only S-opsin Expressing

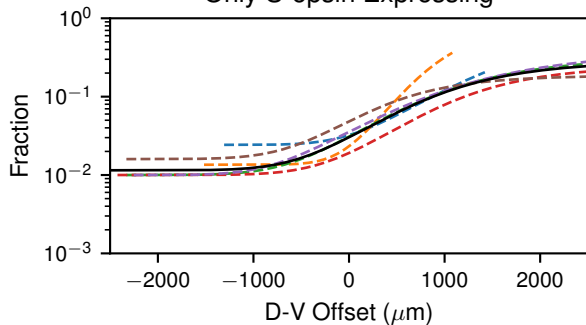

M-opsin

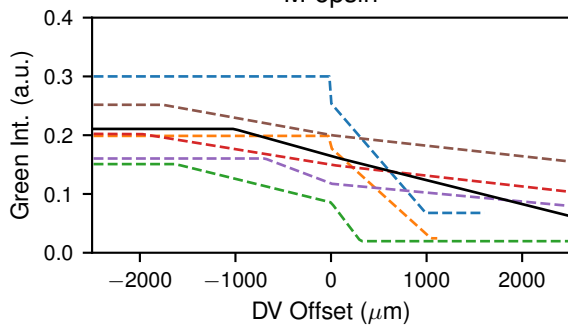

S-opsin

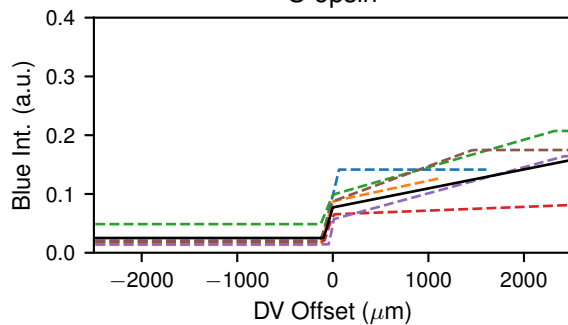

Supplement: S14 Fig — Comparison of the fits for individual retinas (dashed lines) with our hypothetical mean retina used for model parameterization (solid line) along the D-V axis. The top row shows a comparison of the fraction of cells expressing M- and S- opsin, respectively. The middle row shows the fraction of FD(S) cells. The bottom row shows the mean M- and S-opsin expression intensity, respectively. (PDF) [file pcbi.1007691.s018.pdf]

M-opsin Expressing

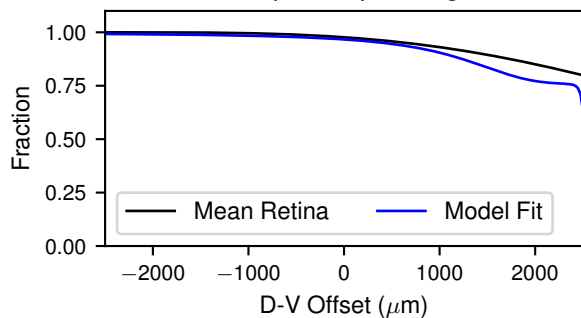

S-opsin Expressing

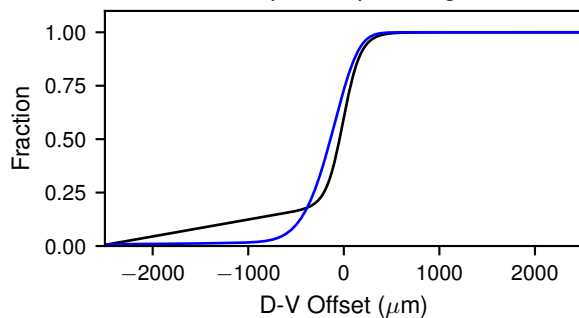

Only S-opsin Expressing

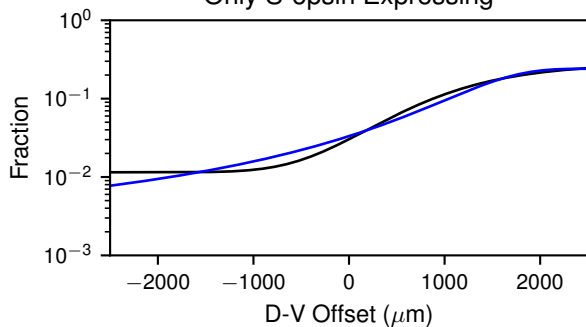

M-opsin

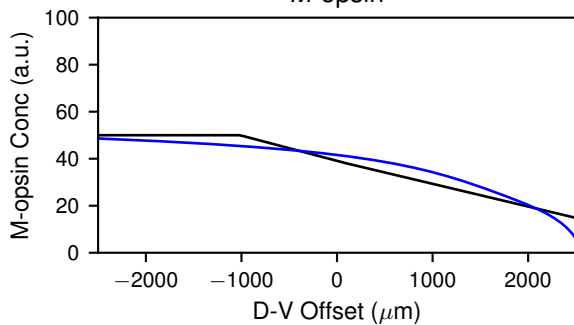

S-opsin

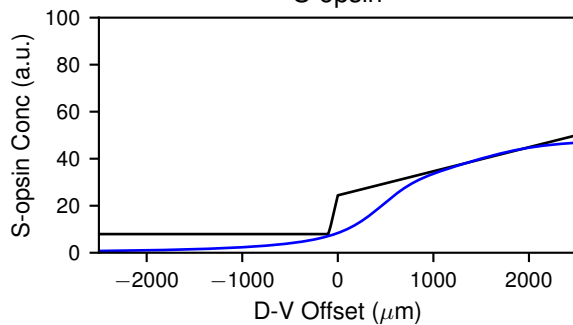

Supplement: S15 Fig — Comparison of the best fit model parameterization (blue) with the hypothetical mean retina (black). The top row shows a comparison of the fraction of cells expressing M- and S- opsin, respectively. The middle row shows the fraction of FD(S) cells. The bottom row shows the mean M- and S-opsin concentration per cell, respectively. (PDF) [file pcbi.1007691.s019.pdf]
