# Supplementary material for: Modeling binary and graded cone cell fate patterning in the mouse retina
Source: PLoS Comput Biol. 2020 Mar 9;16(3):e1007691. doi: 10.1371/journal.pcbi.1007691 (PMC7082072; doi:10.1371/journal.pcbi.1007691)
Supplement: S9 Fig — Mean fraction of cells in various cell populations along the D-V axis from numerical simulations of the model. Plots show the mean value computed from 100 independent simulations. (PDF) [file pcbi.1007691.s013.pdf]

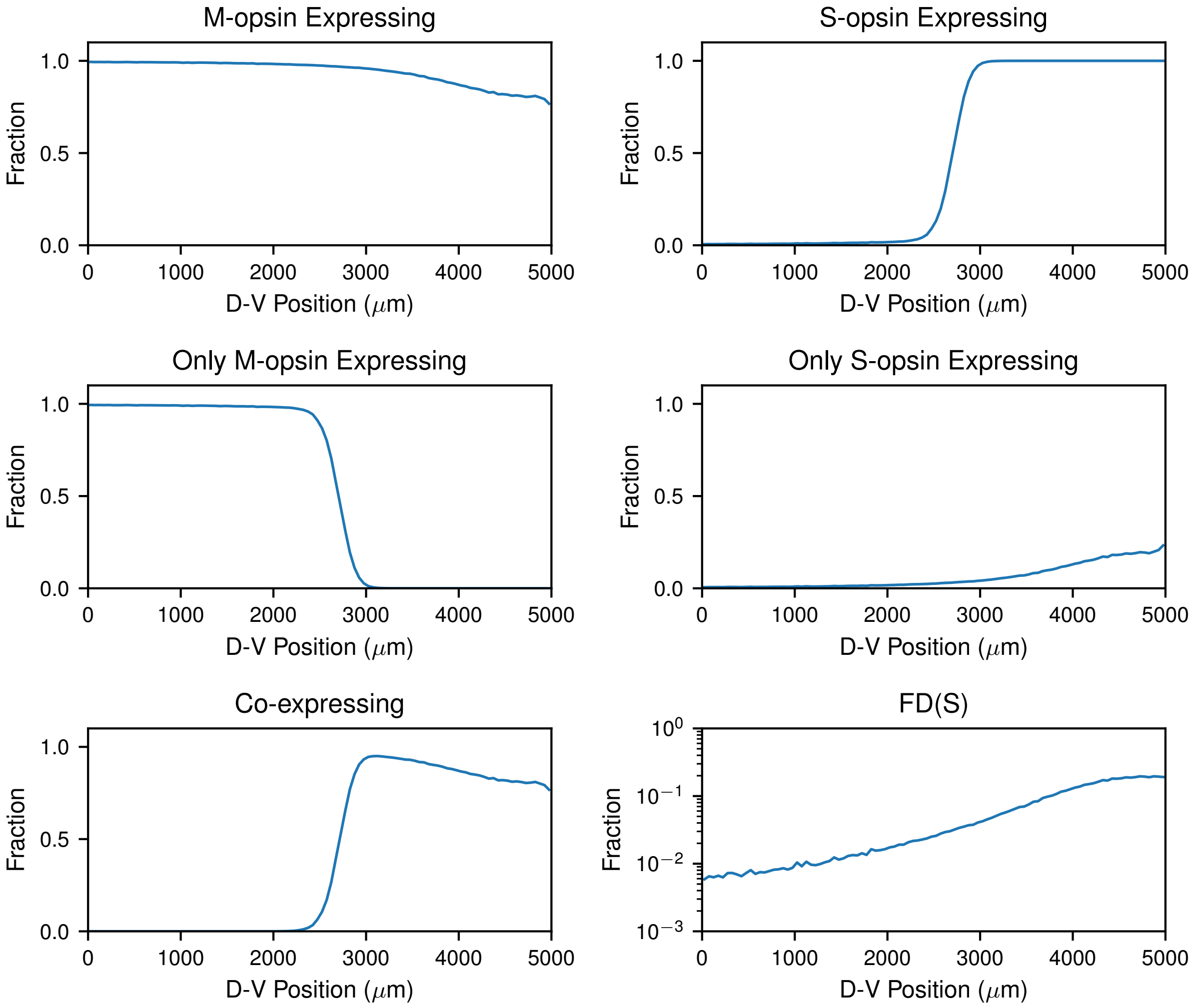
M-opsin Expressing
S-opsin Expressing
Only M-opsin Expressing
Only S-opsin Expressing
Co-expressing
FD(S)
Fraction
D-V Position (μm)
0.0
0.5
1.0
0
1000
2000
3000
4000
5000
10^0
10^{-1}
10^{-2}
10^{-3}
