# Supplementary material for: Modeling binary and graded cone cell fate patterning in the mouse retina
Source: PLoS Comput Biol. 2020 Mar 9;16(3):e1007691. doi: 10.1371/journal.pcbi.1007691 (PMC7082072; doi:10.1371/journal.pcbi.1007691)
Supplement: S3 Fig — Overlap of the fraction of cells expressing (left) M-opsin and (right) S-opsin aligned to the transition midpoint as determined from the S-opsin expression profile. (PDF) [file pcbi.1007691.s007.pdf]

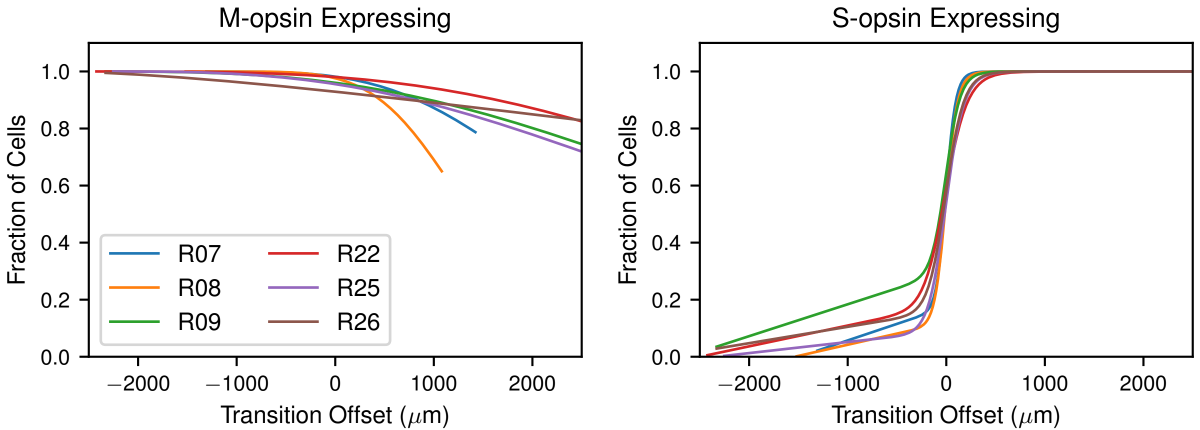

M-opsin Expressing
S-opsin Expressing
Fraction of Cells
1.0
0.8
0.6
0.4
0.2
0.0
−2000
−1000
0
1000
2000
Transition Offset (μm)
R07
R08
R09
R22
R25
R26
