# Supplementary material for: Modeling binary and graded cone cell fate patterning in the mouse retina
Source: PLoS Comput Biol. 2020 Mar 9;16(3):e1007691. doi: 10.1371/journal.pcbi.1007691 (PMC7082072; doi:10.1371/journal.pcbi.1007691)
Supplement: Attachment — Submitted filename: Response.docx [file pcbi.1007691.s020.docx]

MANUSCRIPT PCOMPBIOL-D-19-01633

**Response to Referee Reports**

Thank you to the reviewers for carefully reading through the paper, and for the many helpful and insightful comments. We have addressed each of their concerns. We believe that the manuscript is much improved and ready for publication in PLOS Comput Biol. Below is our detailed response to each of the referees’ points.

**Reviewer 1**

In this manuscript, Eldred et al. analyze the distribution of M- and S-opsin expression in the mouse retina and use an image analysis approach to model the choice of cone cells to express M-opsin, S-opsin, or both. They first characterize in detail the distribution of the two opsins in six wild-type mouse retinas and reproduce what was already known, i.e. that M-opsin is enriched in the dorsal part of the retina, while S-opsin the ventral part. Reasonably, co-expressing cones are mostly enriched in the middle and mid-ventral part. They then argue that their data agree with a previous study (Haverkamp et al 2005), that stated that instead of having three different populations of cone cells (S-expressig, M-expressing, and co-expressing, there exist two population (S-expressing and co-expression competent cones) and pursue to model these two decisions a cone cells has to make: a) a binary decision to express S-opsin or be competent to express both, and b) a graded decision to express different levels of the two opsins, based on their position on the D-V axis. Interestingly, both decisions are influenced by the presence of thyroid hormone T3, whose levels are higher in the dorsal part of the retina and decrease along the D-V axis. Then, they try to show that their model can recapitulate their wild-type data, as well as the reduction of expression of S-opsin in Thrb2 mutant retinas.

While the paper is well done, well written, and interesting to read and the question is definitely very intriguing, I don’t believe that it offers any biological, computational, or conceptual advance to warrant publication in PLoS Computational Biology. The authors repeat what was already known in a quantitative manner and come up with a not-so accurate model, which was already proposed in the past. Moreover, the data that support that their model recapitulates the observed data (i.e. Figures 4, 8, and 9) are the least convincing figures of the manuscript.

We appreciate the referee’s positive comments regarding the writing of the manuscript and the general interest of the work. The novelty of the work lies in the quantitative analysis and modeling. Such pipelines are not readily available/used in developmental biology to study patterning on the scale of the mouse retina. This paper provides a paradigm for studying complex patterns. This point is summarized in the abstract:

*“Using these single-cell data, we developed a quantitative, probabilistic model of cone cell decisions in the retinal tissue based on thyroid hormone signaling activity. The model recovers the probability distribution for cone fate patterning in the mouse retina and describes a minimal set of interactions that are necessary to reproduce the observed cell fates. Our study provides a paradigm describing how differential responses to regulatory inputs generate complex patterns of binary and graded cell fates.”*

To address whether the model recapitulates the observed data, we addressed the reviewer’s specific comments:

In Fig. 4, while I have no reason to disagree that there are two populations of cone cells (S-expressing and co-expression competent), this is not shown or supported by these data. The authors manually draw a white dashed line that agrees with their hypothesis, but they could have as easily drawn another white line between the co-expressing population and the M-expressing population, where there is a dip in the yellow portion of the heatmap (approximately in position (0.15,0.02)). I would be far more comfortable with these data, if the white-dashed line was drawn in a “computational” way, rather than manually.

In Fig. 4, the white line is a computationally optimized split of the density in the high dimensional space. To address the concerns with the line being a subjective interpretation, we included identification of the clusters using another independent density-based clustering algorithm known as HDBSCAN. This method recapitulates the two clusters calculated by our previous analysis. We updated Fig. 4 to show the clusters computed by the method along with a rendering of the high dimensional space. We also added text in the manuscript to describe this new analysis:

**Previously**: *“To refine our classification of cone subtypes, we calculated the joint probability*

*distribution for S- and M-opsin intensity in individual cells for each retina (****Fig. 4A, S4****). When*

*considering all cones in the retina, there appear to be three clusters of cell-types corresponding*

*to the three classifications that we defined earlier: S-only, M-only, and co-expressing. However,*

*when the data are analyzed by D-V position (****Fig. 4B-F, S5****), a different pattern emerges. First,*

*we see an S-only cluster than has high and consistent expression of S-opsin while increasing in*

*abundance along the D-V axis (****Fig. 4****). Interestingly, the other two clusters resolve into a single*

*cluster that changes position in a continuous way, gradually moving from low S-opsin*

*expression and high M-opsin expression in the dorsal region, to moderate S-opsin expression*

*and low M-opsin expression in the ventral reigion (****Fig. 4****). Thus, these data suggest that the*

*mouse retina contains only two main subtypes of cones: 1. S-only cones that have high S-opsin*

*expression independent of D-V position and 2. co-expression competent (CEC) cones that*

*express S- and/or M- opsins dependent upon D-V position.”*

**Updated: *“****To distinguish classes of cone subtypes, we performed a cluster analysis. When considering all cones in the retina, there visually appear to be three groups of cell-types corresponding to the three classifications that we defined earlier: S-only, M-only, and co-expressing. However, when we include the clustering analysis, a different pattern emerges (****Fig. 4A****). Expression levels do not cluster around single points, but rather follow along manifolds in the high dimensional space. We used HDBSCAN, a density-based clustering analysis that connects regions of high local density, to generate clusters* *(Campello et al., 2013). The method identified two distinct clusters of expression (****Fig. 4B****). The two clusters are separated by a region of low density in the high dimensional space.*

*To study the properties of these clusters, we calculated the joint probability distribution for S- and M-opsin intensity in individual cells for each retina (****Fig. 4C, S4****). First, we see an S-only cluster that has high and consistent expression of S-opsin while increasing in abundance along the D-V axis (****Fig. 4D-H, S5****). Interestingly, the other cluster changes position in a continuous way, gradually moving from low S-opsin expression and high M-opsin expression in the dorsal region, to moderate S-opsin expression and low M-opsin expression in the ventral region (****Fig. 4D-H, S5****).”*

In Fig. 8, while the authors argue that their model recapitulates their observations, in fact now the difference between S-opsin expressing cells and M-opsin expressing cells is now more evident. I would have loved to seen the equivalent of Figure 4A in Figure 8, as well. I believe that if the authors overlay Figures F-J, there will be no “dip” between M-opsin expressing cells and co-expressing cells (i.e. the yellow portion will be continuous). This would contradict the observed data and will also justify my previous comment about how the white dashed line was drawn.

We thank the referee for their suggestions for making Figure 8 more clear. We have added the overlaid probability density of all cells for the simulated data to Figure 8 as the referee suggested. As expected, there is a dip between the M-opsin and co-expressing cells. This is an inevitable property that results from the manifold that the expression states follow, which we have added to Figure 4. The direction followed by the manifold changes very quickly when moving along the graded expression of S- and M-opsin. Thus, cells build up into two high-density regions when projected onto two dimensions.

This is also illustrated by the fact that in Figures 8F-J, the yellow (“hot”) region doesn’t even “approach” the white dashed line, while in Figures 8D-E, it almost overlaps with it. Again, I am not disagreeing with the biology, rather with the interpretation that the model recapitulates observed data.

The difference between the spread of the clusters in panels A-E and F-J is due to the increased variance in the experimental data compared to the model. The referee is correct that the cluster is wider in the experimental column. However, the colors are log-scaled, such that the amount of density approaching the white line is actually quite small, on the order of 0.01% of the total population. We made this clearer in the figure caption and, together with the additional cluster analysis described above in Figure 4, we feel that the data and model are in quite good agreement.

**Updated, text added to figure legend page 18:**

**Figure 4, C-H) *“****Cones are ranked according to the intensity of S- and M-opsin expression levels. Intensity values are represented in arbitrary units. Each point is colored according to the log_10_[Probability] of expression levels. A line is drawn on the graph to show the separation between the two discrete populations of S-opsin only and CEC cone populations.”*

This becomes even stronger in Figure 9, where the authors argue that experimental and simulated data agree with each other, while this is definitely not is shown in this figure.

We performed additional simulations that better account for the retina-to-retina variability that is observed in the experimental data. Using these new simulations, we have updated Fig. 9. We feel that Fig. 9 now shows that once experimental variability is accounted for, the model and experimental data are in good agreement. We have also updated the text to describe our new modeling.

**Previously:** *“In our model, they are coupled through the T3 gradient and we wanted to test if the experimental retinas displayed more or less variability than expected. As both the experimental data and our probabilistic model display retina-to-retina variability, we checked for overlap of the corresponding probability distributions.*

*We calculated the probability of having a given CEC population fraction at the S-opsin transition point from both our simulations and experimental retinas (****Fig. 9A****). We also calculated the rate at which the CEC population decreases at the transition point (****Fig. 9B****). As can be seen from both plots, the two probability distributions are in good agreement.”*

**Updated: “***In our model, they are coupled through the T3 gradient and we wanted to test if the experimental retinas were also coupled. As the experimental data show large retina-to-retina variability, we performed 100 additional simulations with parameters sampled from normal distributions parameterized using the experimental variance and checked for overlap of the corresponding probability distributions for two observables.*

*First, we calculated the probability of having a given CEC population fraction at the S-opsin transition point from both our simulations and experimental retinas (****Fig. 9A****). Second, we calculated the rate at which the CEC population decreases at the transition point (****Fig. 9B****). As can be seen from both plots, the experimental and simulated probability distributions are in good agreement.”*

**Reviewer 2**

**Reviewer comment**

The manuscript by Eldred et al. offers fresh views and approaches to understanding cell fate decisions, using a highly accessible system within the mouse retina: patterns of expression of M vs. S (vs. both) opsins. While the roles of TRb2, and gradients of T3 for patterning M vs. S expression have been known for some time, the current study and analysis provides finer detail and an interesting new model – advancing the concept of a binary and then graded cell fate. This study appears to be appropriate for PLOS Computational Biology, and is mostly written in a manner that is understandable by a broad audience (not strictly a computational audience). However, some key methods information is lacking, there is room for clarification in several places, and the manuscript misses an opportunity to compare with existing developmental literature.

We appreciate the referee’s positive comments regarding the subject matter, broad accessibility of the manuscript, and suitability for publication in PLoS computational Biology. We agree that our analysis provides finer detail of the mouse retinal patterning, and our model advances the concept of binary and graded cell fate decisions. We also thank the referee for their suggestions that enabled us to clarify the methods and provide a comparison to existing developmental literature. We describe these changes in detail:

**Specific comments**

Abstract. Within the abstract, the authors use the term “stochastic model,” and while this refers to their tool for estimating probability distributions, it is also confusing because there are a number of “stochastic models” for cone fate decisions out there in the retina world, and these models mean something very different from the binary + graded paradigm the authors propose. This reviewer suggests simply striking out the word “stochastic” from the abstract to avoid this confusion.

To address the referee’s concerns, we substituted the word “probabilistic” for “stochastic” everywhere in the manuscript where we refer to the modeling methodology, and specifically in the abstract to address this concern. We provide one example of a change from the abstract here:

**Previously**: *“Using these single-cell data we developed a quantitative, stochastic model of cone cell decisions in the retinal tissue based on thyroid hormone signaling activity.”*

**Updated:** *“Using these single-cell data we developed a quantitative, probabilistic model of cone cell decisions in the retinal tissue based on thyroid hormone signaling activity.*

Page 5, 1st paragraph. Superimposed upon the perfect grid of the zebrafish cone mosaic is variable patterning of opsin expression within the L and M populations.

We agree with the reviewer. We have removed the mention of the perfect grid of the zebrafish cone mosaic and focus on the highly variable pattern in the mouse retina that we analyze.

**Previously:** *“The near perfect grid of cone subtypes in the zebrafish eye is in stark contrast with the variable patterning of cone subtypes in the D-V axis of the mouse retina…”*

**Updated:** *“The highly variable arrangement of cone subtypes in the D-V axis of the mouse retina provides a paradigm to develop computational approaches that describe complex patterns of cell types across a tissue”*

Page 5, 2nd paragraph. Upon reading this section, this reviewer expected the manuscript to contain developmental studies to back up the predicted two-step cone patterning process. The authors should be clear here that the model is based upon analysis of adult retina, and is predictive of development.

We have modified the text to emphasize that the focus of this work is computational analysis and modeling. We now clearly state that these studies address the adult retina and are predictive of development.

**Previously:** *“Here, we present a multiscale model describing the emergence of the complex arrangement of cone cells found in the mouse retina using both stochastic and deterministic methods. We collect data for the model from analysis of immunofluorescence images of retina tissues to identify and map individual cones along the entire D-V axis of the mouse retina. Based on opsin expression in the individual cells, we find that cones can be classified into two main subtypes: S-only cones and co-expression competent (CEC) cones.”*

**Updated:** *“Here, we present a multiscale computational model describing the emergence of the complex arrangement of cone cells found in the adult mouse retina using both probabilistic and deterministic methods. We collect data for the model from analysis of immunofluorescence images of adult retina tissues to identify and map individual cones along the entire D-V axis of the mouse retina. Based on opsin expression in the individual cells, we find that terminally differentiated cones can be classified into two main subtypes: S-only cones and co-expression competent (CEC) cones.”*

Page 5, bottom paragraph. Here and in the Methods, the authors should state the age(s) of animals that were sampled. This information is not clear in the Methods, and readers need to know this at the outset of the Results.

We understand the referee’s concerns regarding the ages of the mice used for the study. We have provided the reader with the ages of the mice in the beginning of the results section. We have also modified the methods section to make the ages of the mice used clear.

**On page 5, Results**

**Previously:** *“To globally characterize patterning of opsin expression in the mouse retina, we first examined the relative intensity of S- and M-opsin expression in the D-V and temporal-nasal (T-N) axes at low resolution for whole-mounted retinas.”*

**Updated:** *“To globally characterize patterning of opsin expression in the adult mouse retina at 2 to 8 months old, we first examined the relative intensity of S- and M-opsin expression in the D-V and temporal-nasal (T-N) axes at low resolution for whole-mounted retinas.*

**In the Methods section**

**Previously:** “*Male and female mice at 2–8 months of age were housed in plastic translucent cages with steel-lined lids in an open room... All animals were handled in accordance…”*

**Updated:** “*Male and female mice were housed in plastic translucent cages with steel-lined lids in an open room… Retinal dissections were performed on mice at 2–8 months old. All animals were handled in accordance…”*

Page 9, bottom. Was the deterministic diffusion equation informed at all by actual data, or at least consistent with what is known? Roberts et al. (2006) reports dorsal and ventral T3 levels from retinas collected at P0, P4, and P11.

We thank the referee for their interest in the deterministic diffusion equation and discussion of Roberts et al (2006). The deterministic diffusion equation presented in the manuscript is based on relative T3 levels. Roberts et al. (2006) reports a differential gradient in [T3] and [T4] between the dorsal and ventral regions of whole retina samples. However, they do not provide intracellular concentrations of T3 specifically in photoreceptor cells. For this reason, we built the model using relative values for [T3], assuming that the concentration is 100 times greater at the dorsal edge (Table S3), which is not inconsistent with any known data. . We would like to point out that knowing the specific values will not change the model we have built, but rather add biological parameters for the receptor activity.

We have updated the text to clarify this point here:

**Previously: *“****Within the modeled volume, T3 molecules diffuse according to the deterministic diffusion equation with constant concentration boundaries, establishing a D-V gradient (****Eq. S1, SI Methods****).”*

**Updated*:*** *“Within the modeled volume, T3 molecules diffuse according to the deterministic diffusion equation with constant concentration boundaries, establishing a D-V gradient.* *Roberts et al. (2006) reports a differential gradient in [T3] and [T4] between the dorsal and ventral regions of whole retina samples, however, it is not known what the intracellular concentrations of T3 are specifically in photoreceptor cells at a single cell level* *(Roberts et al., 2006). For this reason, we use relative values for [T3] to build a deterministic diffusion equation (****Eq. S1, SI Methods****).”*

Page 9, bottom (and Fig. 6). The authors use the term “stochastically” again in a somewhat confusing/misleading manner, particularly because the “stochastic” progression is influenced by a diffusible ligand binding to its receptor. The component of the model shown in Fig. 6B(2) does not look stochastic.

To address the referee’s concerns, we removed word “stochastic” when referring to the model. We changed the word “stochastic” to “random” when we describe cellular activity.

**Previously:** *“Also, within the volume, we stochastically modeled ~23,000 individual cones spaced on a hexagonal grid (Fig. 7). These cells stochastically exchange T3 molecules with the surrounding deterministic microenvironment. Within each cone, T3 can bind to and activate Thrβ2 (Thrβ2*), controlling both fate specification and opsin expression (Fig. 6B1-4).*

*For the binary fate decision, we defined a fate determinant function, FD(X). Photoreceptors start in an undifferentiated fate, FD(U), and stochastically progress to either the FD(S) (S-only) fate or FD(C) (CEC) fate (Fig. 6B2).”*

**Updated:** *“Also, within the volume, we modeled ~23,000 individual cones spaced on a hexagonal grid (Fig. 7). These cells randomly exchange T3 molecules with the surrounding deterministic microenvironment. Within each cone, T3 can bind to and activate Thrβ2 (Thrβ2*), controlling both fate specification and opsin expression (Fig. 6B1-4).*

*For the binary fate decision, we defined a fate determinant function, FD(X). Photoreceptors start in an undifferentiated fate, FD(U), and progress to either the FD(S) (S-only) fate or FD(C) (CEC) fate (Fig. 6B2).”*

Page 11, bottom. The authors refer to their stochastic simulations, and again the word is unfortunately misleading (even if accurately used). Perhaps here, or earlier in Results, the meaning of stochastic modeling/simulations could be defined?

To address the referee’s concern, we substituted the word “probabilistic” for “stochastic” when we refer to the modeling methodology, and specifically on page 7 to address this concern. We also included other instances throughout the manuscript where we changed “stochastic” to “probabilistic”.

**Previously:** *“Because stochasticity in both the experimental data and our model introduces retina-to-retina variability, we checked for overlap of the corresponding probability distributions… In particular, the widths of the experimental probability distributions are similar to the widths from the stochastic simulations.”*

**Updated:** *“As the experimental data show large retina-to-retina variability, we performed 100 additional simulations with parameters sampled from normal distributions parameterized using the experimental variance and checked for overlap of the corresponding probability distributions… In particular, the widths of the experimental probability distributions are similar to the widths from the probabilistic simulations..”*

**Previously (p.4, bottom): *“****Multiscale approaches that combine stochastic and deterministic models of tissues at the scale of individual cells have shown promise in helping to elucidate the details of tissue patterning…”*

**Updated: *“****Multiscale approaches that combine probabilistic and deterministic models of tissues at the scale of individual cells have shown promise in helping to elucidate the details of tissue patterning…”*

**Previously (p.13, bottom): *“****We developed a mathematical model that described both the binary fate specification process of cones and the graded expression of opsins, all driven by an external gradient. Stochastic modeling of this complex process generated probability distributions that we used to compare with experimentally observed cell distributions to test hypotheses about the connections between cell fates. Stochastic modeling is now sufficiently mature to perform detailed simulations of tissue-level cell-fate decisions. Combining stochastic models with high-throughput microscopy is a powerful tool for helping to understand complex relationships in tissues.”*

***Updated: “****We developed a mathematical model that described both the binary fate specification process of cones and the graded expression of opsins, all driven by an external gradient. Probabilistic modeling of this complex process generated probability distributions that we used to compare with experimentally observed cell distributions to test hypotheses about the connections between cell fates. Probabilistic modeling is now sufficiently mature to perform detailed simulations of tissue-level cell-fate decisions. Combining probabilistic models with high-throughput microscopy is a powerful tool for helping to understand complex relationships in tissues.”*

**Previously (p.16, second to last paragraph):** *“Multiscale modeling of the retina strip was performed using a hybrid deterministic-stochastic method. Diffusion of T3 in the microenvironment of the retinal strip was modeled using the diffusion partial differential equation (PDE). The PDE was solved using an explicit finite difference method. The cone cells were modeled using the chemical master equation (CME) to describe the stochastic reaction scheme implementing the cell fate decision-making. The CME for each of the 23,760 cone cells was independently sampled using Gillespie’s stochastic simulation algorithm…”*

**Updated: *“****Multiscale modeling of the retina strip was performed using a hybrid deterministic-probabilistic method. Diffusion of T3 in the microenvironment of the retinal strip was modeled using the diffusion partial differential equation (PDE). The PDE was solved using an explicit finite difference method. The cone cells were modeled using the chemical master equation (CME) to describe the probabilistic reaction scheme implementing the cell fate decision-making. The CME for each of the 23,760 cone cells was independently sampled using Gillespie’s probabilistic simulation algorithm…”*

Page 13. The authors should compare their predictions with what is known of the developmental time-course of M and S opsin patterning (and formation of the T3 gradient) within the literature. Do the two-step results bear out experimentally, or are they at least consistent with the reports from Roberts et al. (2006) and Aavani et al. (2017), among others? If not, how could any conflicts be resolved by either modifying the model or performing experiments?

We thank the referee for their suggestions to broaden the scope of our paper to include a discussion of developing mouse retina, and how this compares to our model. We have added a paragraph to the discussion section to elaborate on this topic.

**We have added the following paragraph to the discussion on page 13, paragraph 2:**

*“Our study models how the terminal pattern of opsin expression and cone subtypes can be regulated by thyroid hormone signaling. A next step is to address the temporal dynamics of this process during development. Thyroid hormone signaling through Thrβ2 is necessary and sufficient to induce M-opsin and inhibit S-opsin* *(Ng et al., 2001; Roberts et al., 2006). S-opsin mRNA is expressed highly in the ventral region and M-opsin mRNA is expressed highly in the dorsal region at P0* *(Aavani et al., 2017). Interestingly, distinct differences in T3 levels conducted on dorsal and ventral halves are only observed by P10* *(Roberts et al., 2006). These observations suggest two main possibilities. First, earlier differences in T3 levels may be cell-type-specific and/or below the threshold for detectability and these subtle differences in TH establish the cone subtype pattern. Second, a two-step mechanism controls patterning whereby opsin expression is (1) initially patterned by a TH signaling independent pathway and then (2) maintained and/or reinforced by TH signaling to determine the terminal pattern. As TH signaling is necessary and sufficient to determine the terminal pattern, our model is consistent with either of these possibilities.”*

Also missing from the discussion is a mention of the interesting outcome of knocking out S opsin (Daniele et al., 2011) – M opsin expression increases, speculatively due to reduced competition for translational machinery. This outcome suggests factors other than those offered in the current manuscript can regulate opsin expression levels.

We thank the author for this helpful suggestion. It is particularly interesting to note that the results of the Daniele et al. 2011 manuscript corroborate the two classes of cone cells: S-opsin only expressing cells, and co-expression competent cells. Daniele et al. 2011 found that S-opsin knock out mice have a significant number of cone cells in the ventral region that do not express any opsin and have disrupted cone morphology. We hypothesize that this degrading cone cell population is the same population that we describe in our manuscript as S-only cones. In the cells in the mid and dorsal retinal regions, the M-opsin protein levels are higher than in wild type mice, presumably due to less competition for translation machinery, and therefore higher translation of M-opsin mRNA transcripts. We have added a discussion of these ideas:

**Paragraph added to page 13 paragraph 3:**

*“Additional developmental studies support our model for cone fate specification. Daniele et al. 2011 studied the effects when S-opsin was knocked out. Mice lacking S-opsin have a significant number of cone cells in the ventral region that do not express any opsin and have disrupted cone morphology. We hypothesize that this degrading cone cell population is the S-only cones. In the cells of the mid and dorsal retinal regions, the M-opsin protein levels are higher than in wild type mice, presumably due to less competition for translation machinery, and therefore higher translation of M-opsin mRNA transcripts. In addition, other factors that modulate opsin levels in could be added to the model, such as RNA transcript availability* *(Daniele et al., 2011)”*

Page 15. Probably the antibodies were polyclonals, but this should be stated.

We thank the referee for this suggestion, and have addressed this concern by clearly stating in the methods that the antibodies used were polyclonal.

**Previously:** “*Primary antibodies were used at the following dilutions: goat anti-SW-opsin (1:200) (Santa Cruz Biotechnology), rabbit anti-LW/MW-opsins (1:200) (Millipore)*.”

**Updated:** “*Primary antibodies were used at the following dilutions: polyclonal goat anti-SW-opsin (1:200) (Santa Cruz Biotechnology), polyclonal rabbit anti-LW/MW-opsins (1:200) (Millipore)*.”

Page 16. Do the authors mean outer segment boundaries rather than cell boundaries?

We thank the referee for their careful reading of the methods. We meant outer segment boundaries of the cone cells and modified the methods to reflect this clarification. We also referred to the “cell” boundaries rather than “outer segment of cone cell” boundaries in the main text. Based on the referee’s suggestion, we also modified the main text to contain more precise language regarding cell measurement.

**Methods section**

**Previously:** *“Finally, cell boundaries were reconciled across both channels to obtain a complete list of identified cone cells. Full details are given in the SI Methods.”*

**Updated:** *“Finally, the outer segment boundaries of cone cells were reconciled across both channels to obtain a complete list of identified cells. Full details are given in the SI Methods.”*

**Main text**

**Previously:** *“We developed an analysis pipeline to identify the position, size, and boundaries of each cell, a process known as segmentation (see* ***SI Methods, 1.1.1****). Overall, we identified ~250,000 total cells across six retinas. Using these boundaries, we calculated the expression intensity of M- and S-opsin for each cell. We classified cones into groups expressing M-opsin only (****Fig. 2E, K, Q****), S-opsin only (****Fig. 2F, L, R****), and S- and M-opsin co-expression (****Fig. 2G, M, S****). The pipeline did not identify distinct morphological or size differences among the cone (data not shown). We found that the pipeline’s accuracy and false positive rate were comparable to hand-scored retinas (see* ***SI Methods 1.1.2****).*

*After obtaining the cell boundaries, we quantified the density of cone subtypes based on opsin expression relative to D-V position.”*

**Updated:** *“We developed an analysis pipeline to identify the position, size, and boundaries of the outer segment of each cone cell, a process known as segmentation (see* ***SI Methods, 1.1.1****). Overall, we identified ~250,000 total cells across six retinas. Using these outer segment boundaries, we calculated the expression intensity of M- and S-opsin for each cell. We classified cones into groups expressing M-opsin only (****Fig. 2E, K, Q****), S-opsin only (****Fig. 2F, L, R****), and S- and M-opsin co-expression (****Fig. 2G, M, S****). The pipeline did not identify distinct morphological or size differences among the cone outer segments (data not shown). We found that the pipeline’s accuracy and false positive rate were comparable to hand-scored retinas (see* ***SI Methods 1.1.2****).*

*After obtaining the outer segment boundaries of the cone cells, we quantified the density of cone subtypes based on opsin expression relative to D-V position.”*

Figure 9 took a while to appreciate, and may be easier for a reader to grasp if the X axes had slightly greater scales. As presented, the first impression is that the experimental and simulated positions and slopes do not map on to each other very well.

We thank the referee for their helpful suggestion for making this figure more clear. We have performed additional simulations and updated Fig. 9 in response to this comment and that of another reviewer. Fig. 9 now presents a comparison of experimental and simulated data in which experimental retina-to-retina variability has been better taken into account. We feel that there is now a good overlap between the experiment and the model. The model distributions are now approximately the same width. We have also expanded the x scale as suggested by the reviewer. The text has been updated to describe our new simulations.

**Previously:** *“In our model, they are coupled through the T3 gradient and we wanted to test if the experimental retinas displayed more or less variability than expected. As both the experimental data and our probabilistic model display retina-to-retina variability, we checked for overlap of the corresponding probability distributions.*

*We calculated the probability of having a given CEC population fraction at the S-opsin transition point from both our simulations and experimental retinas (****Fig. 9A****). We also calculated the rate at which the CEC population decreases at the transition point (****Fig. 9B****). As can be seen from both plots, the two probability distributions are in good agreement.”*

**Updated: “***In our model, they are coupled through the T3 gradient and we wanted to test if the experimental retinas were also coupled. As the experimental data show large retina-to-retina variability, we performed 100 additional simulations with parameters sampled from normal distributions parameterized using the experimental variance and checked for overlap of the corresponding probability distributions for two observables.*

*First, we calculated the probability of having a given CEC population fraction at the S-opsin transition point from both our simulations and experimental retinas (****Fig. 9A****). Second, we calculated the rate at which the CEC population decreases at the transition point (****Fig. 9B****). As can be seen from both plots, the experimental and simulated probability distributions are in good agreement.”*

**Reviewer 3**

The manuscript titled "Modeling binary and graded cone cell fate patterning in the mouse retina" presents image analysis and computational modeling results that show how photoreceptor cells in the mouse retina develop and differentiate.

This paper is extremely well written, their conclusions are well support by their data, and all of their material is publicly available for reproduction of their results. I see no reason that this paper should not be published immediately.

We thank the referee for their enthusiastic and positive feedback on the manuscript and their recommendation for publication in PLoS Computational Biology.

**Editorial Changes**

We have also made the following editorial changes to follow journal guidelines:

1. We have split all Text, Tables, and Figures from the supporting information into separate files. We have added legends for each file to the main manuscript.

2. We have removed a reference to “data not shown” on page 6 in the main manuscript.

**Previously:** *“The pipeline did not identify distinct morphological or size differences among the cone outer segments (data not shown).*”

**Previously:** *“The pipeline did not identify distinct morphological or size differences among the cone outer segments.”*
