# Supplementary material for: Modeling binary and graded cone cell fate patterning in the mouse retina
Source: PLoS Comput Biol. 2020 Mar 9;16(3):e1007691. doi: 10.1371/journal.pcbi.1007691 (PMC7082072; doi:10.1371/journal.pcbi.1007691)
Supplement: Attachment — Submitted filename: Response.docx [file pcbi.1007691.s021.docx]

MANUSCRIPT PCOMPBIOL-D-19-01633

**Response to Referee Reports**

Thank you to the reviewers for the positive notes on our revisions. We have addressed the final concern regarding the proposed supplementary figure.

**Reviewer 2**

**Reviewer comment**

The manuscript has improved, thanks to the authors for their careful attention to responding to all reviewer comments and suggestions.

Only one comment/concern: The removal of (data not shown) does not seem the best way to respect the journal's requirements. The outcome stated on page 6, that no morphological or size differences were identified, can certainly be shown as a supplemental figure, or added to an existing supplemental figure.

We have added a new supplementary figure showing the data regarding the morphological/size differences between the outer segments of cell expression classes.

**Updated:** We have added new figure S1 in the supplementary materials and renumbered the existing supplementary figures and updated their references and captions.

**Updated:** We have changed the main text to be: *“The pipeline did not identify distinct morphological or size differences among the cone outer segments (Fig. S1).”*

**Updated:** We have added the following figure caption for S1: *“Figure S1: Outer segment size by expression class. The probability distribution for the outer segment area is shown for each expression class. Columns shows different expression classes, as labeled. Rows show different retinas (RXX). No systematic differences were observed between the classes, but note that retina-to-retina variability in outer segment area is present due to variations in mounting and subsequently the angle of image capture for cone cell outer segments.”*
